# Real-world effectiveness of a widely available digital health program in adults reporting a lifetime diagnosis of ADHD

**DOI:** 10.1038/s44184-025-00157-3

**Published:** 2025-08-22

**Authors:** Allen M. Osman, Kevin P. Madore, Paul I. Jaffe, Emanuela Offidani, Ann C. Childress, Jeffrey H. Newcorn, Robert J. Schafer

**Affiliations:** 1 Department of Research and Development, Lumos Labs, San Francisco, CA USA; 2 Department of Psychology, Stanford University, Stanford, CA USA; 3 Clinical Epidemiology and Evaluative Science, Weill Cornell Medicine, New York, NY USA; 4 Center for Psychiatry and Behavioral Medicine, Inc., Las Vegas, NV USA; 5 Department of Psychiatry and Department of Pediatrics, Icahn School of Medicine at Mount Sinai, New York, NY USA

**Keywords:** ADHD, Therapeutics, Outcomes research, Translational research

## Abstract

We examined real-world evidence on whether Lumosity, a remote digital health technology designed to deliver cognitive training to healthy adults, can improve cognition and reduce inattention in adults who reported having received a prior (lifetime) diagnosis of ADHD. Over the course of Lumosity training, this cohort of commercial users was assessed repeatedly online with a neuropsychological test battery (NCPT) and a scale of attention and mood in real-world contexts (BAMS-7). More Lumosity training between successive assessments led to greater improvements on the NCPT composite measure and the attentional subscale of the BAMS-7. This positive dose-response relation was found for six of eight NCPT subtests and three of four BAMS-7 attentional items. Additional findings support the participants’ clinical status and sensitivity of the assessments to ADHD symptoms. These findings provide evidence of cognitive and attentional benefits in a real-world cohort of adults reporting a lifetime diagnosis of ADHD from training with Lumosity under real-world conditions.

## Introduction

The 21st Century Cures Act highlights the value of real-world evidence (RWE) based on real-world data (RWD) obtained outside of traditional clinical trials^1^. RWD from digital health technologies provides many opportunities, including the examination of their therapeutic efficacy within large, diverse cohorts under naturalistic conditions. Digital health technologies have shown promise for improving cognitive outcomes in ADHD^2^, but their efficacy has so far been little studied in adults or under real-world conditions. Here we report RWE showing that Lumosity, a remote digital health technology designed to deliver cognitive training (CT) to the general public, can improve cognition and reduce inattentive symptoms in a large group of adult users who reported having received a prior (lifetime) diagnosis of ADHD.

ADHD is a common, chronic condition marked by patterns of inattention, hyperactivity-impulsivity, or both^3^. It is a disorder that has impacts across the lifespan, often beginning in childhood but persisting through adulthood^4^. The 2016 National Survey of Children’s Health estimates that 6.1 million (9.4%) children aged 2-17 in the U.S had been diagnosed with ADHD^5^. Adult ADHD remains an underdiagnosed condition^6^ and prevalence estimates in adults vary considerably. But the National Institute of Mental Health^7^ estimates the prevalence at 4.4% and slightly higher for males (5.4%) than females (3.2%). Recent studies show a significant increase in prevalence and incidence of adult ADHD over the past 10 years^8^.

The majority of individuals with ADHD in childhood have been found to have symptoms that persist into adulthood^9,10^. Adults may experience inattention (e.g., difficulty staying on task, sustaining focus), hyperactivity (though less than children and sometimes presenting as restlessness), and impulsivity, among others^4,11^. Both childhood and adult ADHD include symptoms consistent with impaired executive functions^12,13^. Executive functions are important for the self-regulation of cognition, affect, and behavior. They include sustained and selective attention, visuospatial and verbal working memory, goal-directed planning, response inhibition, and emotion regulation^12,13^. Imaging studies have shown that ADHD is associated with alterations of the prefrontal cortex, which plays a central role in the neural systems underlying executive functions. Forgetfulness, distractibility, impulsivity, perseveration, and disorganization are all symptoms associated with dysfunction of the prefrontal cortex^14^.

The most common form of treatment for both adult and childhood ADHD involves medications, most often stimulants^15,16^. Because pharmacological treatments may not be completely effective or may produce undesirable side effects, they are often replaced or supplemented with other forms of treatment^17^. The most common non-pharmacological treatments for adult ADHD include behavioral therapies, e.g., cognitive behavioral therapy or psychoeducation^18,19^. But barriers to access may limit the utility of behavioral therapies, as several require administration by trained mental health specialists. Digital therapeutics could help circumvent these pharmacological and non-pharmacological limitations or augment treatment. Of most relevance here are those that employ CT to treat ADHD.

The idea behind CT is to strengthen targeted cognitive functions by exercising them in an engaging, gamified context. Similar to many pharmacological treatments, CT can be designed to target the prefrontal cortex and executive function deficits. While interest in CT for treating ADHD is not new^20^, until recently the evidence for its efficacy has been mixed^21^. But, in 2020, a randomized active-controlled trial sponsored by Akili Interactive provided strong evidence that a game-based digital therapeutic can improve performance on a neuropsychological test of attention (Test of Variables of Attention (TOVA®)) in pediatric ADHD^2^. In subsequent single-arm (i.e., no control group) studies in pediatric^22^, adolescent^23^, and adult^23^ ADHD populations, improvement following training with the same digital therapeutic was found on the same neuropsychological test of attention as well as on ADHD symptom rating scales and measures of impairment.

These findings for pediatric and adult ADHD, along with prior studies supporting the efficacy of Lumosity in the general public^24,25^, suggest that Lumosity might likewise benefit its adult users with ADHD. Lumosity is a digital health and wellness technology that delivers a program of CT games^26^. Among the features that make Lumosity games an effective form of CT are that they 1) target specific cognitive functions, 2) adjust their difficulty to maintain challenge, 3) provide novel experiences to promote new learning, and 4) are engaging and provide positive reinforcement^26^. Design features and factor analyses ^27^ of these games also suggest that many target executive functions like those implicated in ADHD.

Lumosity games have been played over 8 billion times by more than 100 million registered users. Over the course of training, many of these users have taken online neuropsychological tests and scales of real-world cognition multiple times, as well as reported on a variety of diagnosed medical conditions, including ADHD. Examining de-identified data from these latter users, we report here RWE concerning the effects of Lumosity training on cognitive function and inattentive symptoms in adult ADHD. Participants in the study included users who reported having received a lifetime diagnosis of ADHD, as well as a healthy control group of users reporting no medical diagnoses. Cognitive function was assessed across multiple domains with a validated web-based battery, the NeuroCognitive Performance Test (NCPT)^28,29^. Inattention was assessed with the Brief Attention and Mood Scale (BAMS-7), a validated survey with items that closely correspond to ones on ADHD symptom rating scales^30^ and DSM-5 criteria for the inattentive subtype of ADHD^3^.

## Methods

### Ethics statement

Both the study and release of the de-identified data were determined by WCG IRB (www.wcgirb.com) to be exempt research under 45 CFR § 46.104(d)(4) because 1) participants’ identities could not be readily ascertained, 2) the investigator did not contact participants, and 3) participants would not be re-identified. Use of de-identified data for any purpose is also covered in the privacy policy agreed to by all participants during their registration to use the Lumosity CT program (www.lumosity.com/en/legal/privacy_policy).

### Participants and procedure

Participants were recruited from among Lumosity users who signed up from 3-26-2007 to 3-26-2020 and indicated English as their preferred language. The country of origin was restricted to the US, Canada, Australia, and New Zealand. A subset of these users was invited via email and an in-app prompt to take the NCPT battery. Taking the NCPT was optional and not required for continued use of Lumosity. Some of the recruited participants also took one or more surveys or scales, including the BAMS-7 and ones reporting whether they had ever been diagnosed with any of 32 medical conditions, including ADHD (listed in Supplementary Note 1). Participants were allowed to take the NCPT and BAMS-7 at multiple time points, each separated by at least 10 weeks. Importantly, they could continue to freely play the games included in their subscription, i.e., engage in CT, between assessments.

The analyses reported here are based on deidentified data from a subset of the recruited participants who were 18–90 years old, took the NCPT and/or BAMS-7 at least once, and completed surveys reporting diagnosed medical conditions. Those who reported any condition on a list of severe neuropsychological disorders (Supplementary Note 1) were excluded. To avoid any effects of prior CT on baseline assessments, participants with more than 25 gameplays before their first NCPT and/or BAMS-7 were excluded as well^25^. These selection criteria were met by 143,806 participants who were arranged into cohorts in several alternative ways, depending on which analyses were performed (see Results and Supplementary Note 1).

### Survey of diagnosed medical conditions

As mentioned, all participants in the study completed a survey of diagnosed medical conditions. The survey began with the question “Have you ever been diagnosed with any of the following medical conditions?”, followed by a list of 32 conditions. These included ADHD, eight severe neuropsychological disorders that served as exclusion criteria, and 23 other conditions. Participants were asked to check any of the conditions that applied. All the conditions listed in the survey are shown in Supplementary Table 1.

The information provided by the survey involves self-report and concerns whether a participant had ever been diagnosed with a condition at any time (as opposed to whether they currently had it). Participants indicating ADHD on the survey should therefore be considered as providing a self-report of a lifetime diagnosis (ADHD-SRLD). A similar survey-based determination of ADHD-SRLD has been used by the CDC to assess the prevalence of adult ADHD^31^.

### Cognitive training

Participants used the commercially available Lumosity program, which provided CT via 69 different games (described individually in Supplementary Note 6) across web and mobile apps over the time period in which the study data were collected. (Games were introduced and discontinued over time, so the exact number of available games varied.)

Design features and factor analyses of these games suggest that many target executive functions. Lumosity games are modeled on paradigms used to study specific cognitive functions in the lab or clinic. Based on their primary cognitive demands, they can be organized into seven cognitive domains: Memory, Attention, Flexibility, Problem Solving, Speed of Processing, Math Skills, or Language Skills. The majority of games, however, make demands in multiple cognitive domains (e.g., Speed of Processing and Flexibility, or Attention and Memory). Alternatively, each game can be characterized by its loadings on a set of latent cognitive factors^27^. One factor has its highest scores in games requiring the maintenance of goal-directed information, or the inhibition of inappropriate responses and activation of appropriate ones. Another has its highest scores in games requiring spatial recall, route planning, or integration of visual information from across the visual field.

As members of Lumosity, participants could train whenever and as much as they pleased. They could play any of the games available on their platform. However, to encourage breadth of training, each new day a user logged onto the platform, they were recommended five particular games. A single five-game session typically lasted about 15 min.

### Neuropsychological assessment

The NeuroCognitive Performance Test^28,29^ is a validated, brief, repeatable, web-based cognitive assessment platform that measures performance across multiple cognitive domains. NCPT subtests are digital translations of commonly used neuropsychological assessments, which can be selected and arranged to form customized batteries. The specific battery used here took about 20–30 min to complete and included the eight subtests described below in their order of presentation. (For further details, see Supplementary Note 2).

(1) Arithmetic Reasoning: designed to assess numerical problem-solving ability^32^. Participants are required to respond as quickly and accurately as possible to arithmetic problems written in words (e.g., “Four plus two =”).

(2) Digit Symbol Coding: used to measure speed of processing and memory^33^. Participants enter the number corresponding to the symbol using the key provided at the top of the screen.

(3) Forward Visual Memory Span and (4) Reverse Visual Memory Span: based on the Corsi Blocks tasks^34^ and designed to assess visual short-term and working memory, respectively. Participants are required to recall a sequence of randomized spatial locations in either forward or reverse order.

(5) Trail Making A: used to measure speed of processing^35^. Participants connect the numbers from smallest to largest as quickly as possible.

(6) Trail Making B: used to measure speed of processing and mental flexibility^35^. Subjects connect numbers (from smallest to largest) and letters (in alphabetical order) alternating between the two (i.e., 1 to A to 2 to B to 3 to C, etc.).

(7) Grammatical Reasoning: based on Baddeley’s Grammatical Reasoning Test^36^ and designed to assess cognitive flexibility and reasoning. This subtest requires participants to rapidly and accurately evaluate potentially confusing grammatical statements.

(8) Progressive Matrices: based on established matrix reasoning assessments^37^ and designed to assess problem solving and fluid reasoning.

Each study participant’s score on each subtest during their first and second assessments was scaled using a pre-computed norm table that mapped raw scores to values on a normal distribution with a mean of 100 and SD of 15. The norm tables were calculated separately for each subtest using scores on their first NCPT from a larger set of Lumosity users who met the following criteria: completed the entire test battery, had logged no more than 25 prior gameplays, and reported their age and educational attainment. The ranks of the scores in each table were reweighted to account for demographic differences (age and educational attainment) between the normative dataset and the 2019 US Census Bureau’s American Community Survey (ACS) 1-year Public Use Microdata Sample. Thus, the ranks in the norm tables approximated those from a population with the same demographic composition as that reported in the 2019 ACS Microdata Sample. Finally, based on the percentile of its rank, each score in the norm table for each subtest was converted by inverse-normal transformation to a value in a normal distribution with a mean of 100 and SD of 15. After scaling with the norm tables, the mean of the scaled subtest scores for each participant was used to generate an overall composite score (Grand Index (GI)) on their first and second NCPT assessments using an analogous norm table. For further details, see refs. ^28,29^.

### Assessment of attention and mood

The BAMS-7 is a validated, brief, repeatable scale of real-world attention and mood designed to be self-administered and taken online^30^. Its separate Attention and Mood subscales have been found previously to have convergent validity with related questionnaires and to be influenced by CT^30^. They have also been found to discriminate between healthy individuals and those reporting a lifetime diagnosis of ADHD, Anxiety Disorder, or Depression, with the Attention subscale providing superior classification performance for ADHD and the Mood subscale for Anxiety or Depression^30^. The two subscales were found in the present study to have acceptable internal consistency and reliability (Cronbach’s alpha) in both the healthy controls (*α* = 0.718, 95% CI = 0.713–0.724 for Attention; *α* = 0.745, 95% CI = 0.740–0.751 for Mood) and participants reporting a lifetime diagnosis of ADHD (*α* = 0.701, 95% CI = 0.683–0.718 for Attention; *α* = 0.737, 95% CI = 0.720–0.753 for Mood). The individual items comprising the Attention subscale are compared in Supplementary Note 3 to descriptions of ADHD inattentive symptoms in the DSM-5 and to related items in other validated assessments of ADHD.

The seven items in the BAMS-7 were drawn from a larger survey employed in a study of CT^24,38^. Three concern attention over the past month. For these, participants were asked to rate how often they: 1) lost track of details, 2) misplaced items, and 3) lost concentration. Response options were on a Likert scale: “Never”, “1–2 times during the month”, “1–2 times per week”, “Several times per week”, “Almost every day”, or “N/A.” The remaining four items were about attention and mood over the past week. For these, participants were asked to rate their level of agreement with statements about whether they: 1) had good concentration, 2) felt anxious, 3) were in a bad mood, and 4) felt sad. Response options were on a Likert scale: “Strongly disagree”, “Disagree”, “Neither agree nor disagree”, “Agree”, “Strongly agree”, or “N/A.” Responses to all seven items (except N/A) were recoded as 0-4, with 0 always signifying the most negative option, and reverse scoring where appropriate. Analogous to the NCPT GI, the Attention subscale is the average of the numeric responses to the four items concerning attention, and the Mood subscale is the average of the responses to the three items concerning mood.

### Statistical analyses

Details concerning each individual statistical analysis are presented in the Results section. All were performed using R statistical software^39^, version 4.0.0. Linear regressions employed the R lm function. ANOVAs employed lm and the Anova function in the R car package. Odds ratios were calculated using logistic regression, which employed the glm function (family = binomial). Effect sizes were calculated using the cohen.d function (hedges.correction = TRUE) in the R effsize package. Cronbach’s alphas were calculated using the alpha function in the R psych package. All hypothesis testing involved two tails. Where indicated, the *p* values of multiple family-wise comparisons were adjusted using a Bonferroni correction.

## Results

The results of this study are presented in three sections. The first describes the cohorts analyzed in the remaining sections. Of special interest is how the demographics and comorbidities of our ADHD-SRLD cohort correspond to those found in the ADHD population at large. The second section compares our ADHD-SRLD cohort with healthy controls on baseline measures of our assessments. These comparisons provide additional evidence validating the self-reported ADHD diagnosis, as well as evidence about the sensitivity of our assessments to ADHD symptoms. Results from the first two sections provide a foundation for interpreting those of the third. The third section examines changes from baseline on the assessments following training with Lumosity. These changes provide RWE on the primary question addressed by this study, i.e., whether Lumosity use under real-world conditions can reduce inattentive symptoms and improve cognition in adults with ADHD.

### Analyzed cohorts of Lumosity users included in the study

Participants were arranged into cohorts in several alternative ways, depending on which analyses were performed. The cohorts are shown in Table 1, along with information about their sizes and demographic compositions. The two cohorts at the top of the table (Overall Cohorts) divide all participants in the study into those who did or did not report a lifetime diagnosis of ADHD (ADHD+ vs. ADHD-). They were used in the analyses of demographics and comorbidities (this section). Shown immediately below are the cohorts on which analyses of the baseline assessments were performed (Baseline Cohorts, next section). The four Baseline Cohorts are divided into two pairs (ADHD-SRLD and Healthy Controls). The ADHD-SRLD pair of cohorts included participants who reported a lifetime diagnosis of ADHD, and the Healthy Control pair included participants who reported no diagnosed medical conditions. The cohorts within each pair consisted respectively of participants who took at least one NCPT and participants who took at least one BAMS-7. The remaining two cohorts (Efficacy Cohorts) consisted respectively of individuals who took at least two NCPTs or two BAMS-7s. The Efficacy Cohorts were used to examine the effects of Lumosity on the assessments for individuals reporting an ADHD diagnosis (final section of Results). Further information on how participants were arranged into cohorts can be found in Supplementary Note 1.

**Table 1:** Age, gender composition, and size of each cohort analyzed in the present study

	N Male	N Female	N Not Reported	Total	Mean (SD) Age
Overall Cohorts					
ADHD +	9359 (6.50%)	8779 (6.10%)	1579 (1.09%)	19,717 (13.71%)	40.35 (15.91)
ADHD −	43,608 (30.32%)	71,437 (49.67%)	9044 (6.28%)	124,089 (86.28%)	52.58 (16.41)
Total	52,967 (36.83%)	80,216 (55.78%)	10,623 (7.38%)	143,806 (100%)	50.90 (16.87)
Baseline Cohorts					
ADHD-SRLD					
≥1 NCPT	9109 (47.27%)	8603 (44.65%)	1555 (8.07%)	19,267 (100%)	40.44 (15.92)
≥1 BAMS-7	1452 (49.13%)	1379 (46.66%)	124 (4.19%)	2955 (100%)	39.38 (16.19)
Healthy Controls					
≥1 NCPT	16,211 (36.14%)	26,170 (58.34%)	2471 (5.50%)	44,852 (100%)	50.79 (16.57)
≥1 BAMS-7	11,606 (42.47%)	14,654 (53.62%)	1065 (3.89%)	27,325 (100%)	47.17 (16.95)
Efficacy Cohorts					
ADHD-SRLD					
≥2 NCPT	3182 (44.39%)	3443 (48.03%)	542 (7.56%)	7167 (100%)	44.28 (16.15)
≥2 BAMS-7	326 (42.72%)	411 (53.86%)	26 (3.40%)	763 (100%)	44.33 (16.32)

#### Demographics

Participants in the ADHD+ cohort were younger and more likely to be male than participants in the ADHD- cohort. Note that both the age and gender compositions of each cohort were determined jointly by the demographics associated with their ADHD status in the population at large and by the demographics of Lumosity users. The demographic composition of the entire study cohort was similar to that found in other observational studies of Lumosity users^25^. Overall, more participants reported being female (56%) than male (37%) and the average age was 51. A similar pattern was found for the ADHD- cohort. In contrast, a different pattern was found for the subset of participants who reported having received a lifetime diagnosis of ADHD. The ADHD+ cohort was slightly more likely to be male than female (95% CI [0.509, 0.523]) and considerably younger (40.4 vs 52.6 yo) than the ADHD- cohort (t(143,804) = 97.531, *p* < 0.0001).

Taking into account that it was sampled from a pool of Lumosity users with a sizeable female majority, the gender composition of the ADHD+ cohort is consistent with that of the adult population at large with ADHD. This latter group has been estimated to have more males than females^7^ (but less so than for childhood ADHD). The age difference between the ADHD+ and ADHD- cohorts is to be expected, given when the diagnosis of ADHD began to be widely recognized. It was first included in the DSM-III in the 1980s (as ADD in 1980 and as ADHD in 1987) and used with increasing frequency over the following decades^40^. Note that the percentage of individuals reporting ADHD in our study (13.7%) is greater than that estimated for the population at large (4.4%)^7^. This might be because 1) the participants who reported a lifetime diagnosis of ADHD included adults whose childhood ADHD had resolved, or 2) adults reporting ADHD are more likely than the general population to use Lumosity.

#### Comorbidities

**Fig. 1:**
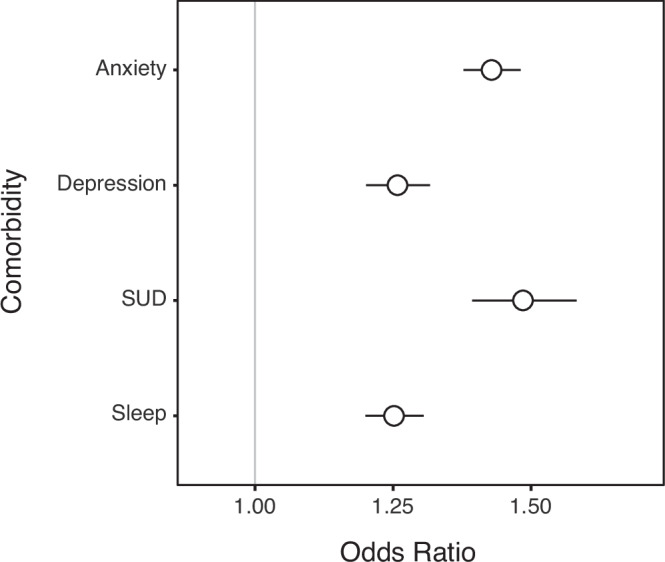
Increased odds of psychiatric conditions associated with ADHD-SRLD. Four psychiatric conditions are shown on the *y*-axis (SUD = Substance Use Disorder). The odds of these conditions given the presence of ADHD-SRLD relative to their odds given the absence of ADHD-SRLD are shown on the x-axis. Bars show 95% confidence intervals.

Participants who reported an ADHD diagnosis also had increased odds of reporting diagnoses of psychiatric conditions found to accompany adult ADHD^7,8,41^. Each of four psychiatric conditions (anxiety, depression, substance use disorder or SUD, and sleep disorders) was examined in a separate logistic regression analysis. The predictor of interest was whether a participant was in the ADHD+ or ADHD- cohort. To control for demographic differences between these cohorts, Age (both linear and quadratic terms) and Gender were included also as predictors in the model. The results of all four analyses are shown in Fig. 1. The y-axis shows each psychiatric condition, and the *x* axis shows how being in the ADHD+ cohort multiplies the odds of reporting it. As can be seen, being in the ADHD+ cohort was associated with increased odds (>1) for all four conditions.

### Baseline differences between ADHD-SRLD and Healthy Control cohorts

Further evidence concerning how well our study identified adults with ADHD, as well as evidence about the sensitivity of our assessments to ADHD symptoms, is provided by the baseline measurements. These were obtained on both the NCPT and BAMS-7 (Methods and Supplementary Notes 2 and 3). For the NCPT, they included scores on each individual subtest, which were combined also to form a GI. For the BAMS-7, they were ratings on the individual scale items, which were combined also into scores on composite Attention and Mood subscales. Baseline measurements for the ADHD-SRLD cohorts were compared to those for the Healthy Control cohorts. To control for demographic differences between cohorts, all estimates and comparisons of baseline measures involved regression models that included Age (both linear and quadratic terms) and Gender as covariates.

**Fig. 2:**
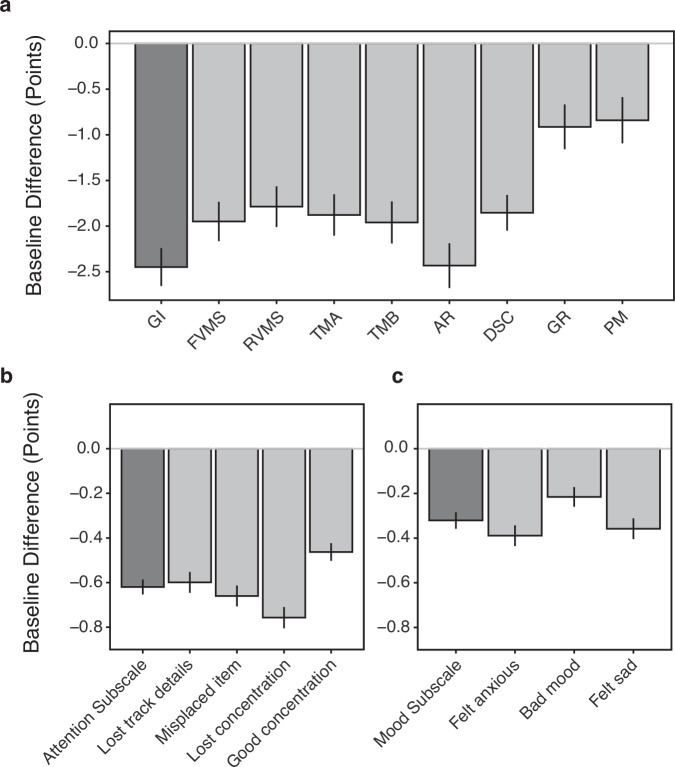
ADHD-SRLD cohorts relative to healthy controls on baseline measures. **a** NCPT; **b** BAMS-7 Attention subscale; **c** BAMS-7 Mood subscale. Dark gray bars show differences between the ADHD-SRLD cohorts and healthy controls (0 on the y-axis) on aggregate measures (Grand Index, Attention subscale, and Mood subscale); light gray bars show differences on the individual NCPT subtests and BAMS-7 items. Negative baseline differences indicate impairment of ADHD-SRLD cohorts relative to healthy controls. Bars show 95% confidence intervals. NCPT measures: GI Grand Index, FVMS Forward Visual Memory Span, RVMS Reverse Visual Memory Span, TMA Trail Making A, TMB Trail Making B, AR Arithmetic Reasoning, DSC Digit-Symbol Coding, GR Grammatical Reasoning, PM Progressive Matrices.

Comparisons between the ADHD-SRLD and Healthy Control cohorts on the baseline NCPT and BAMS-7 are shown respectively in the top and bottom panels of Fig. 2. Differences from the Healthy Control cohorts (horizontal line at 0 on the *y*-axis) on the individual subtests and items are shown by the light gray bars, and differences on the aggregate measures (GI, Attention subscale, and Mood subscale) are shown by the dark gray bars. Negative differences indicate impairment for the ADHD-SRLD cohorts, and error bars show 95% confidence intervals. As can be seen, the ADHD-SRLD cohorts were impaired relative to the Healthy Control cohorts on all measures. Impairments were found on GI (t(64,113) = 23.207, *p* < 0.0001) and all NCPT subtests (t(64,113) > 6.501, *p* < 0.0001 on all eight after Bonferroni correction), on the Attention subscale (t(30,274) = 36.633, *p* < 0.0001) and its individual items (t(30,274) > 22.973, *p* < 0.0001 on all four after Bonferroni correction), and on the Mood subscale (t(30,274) = 17.097, *p* < 0.0001) and its individual items ((t(30,274) > 9.627, *p* < 0.0001 on all three after Bonferroni correction). When considered in terms of effect size, the largest difference between the ADHD-SRLD and Healthy Control cohorts involved inattentive symptoms (Hedges G on Attention subscale = 0.708, 95% CI = 0.670–0.747). Smaller effect sizes were found for impairments of mood (Hedges G on the Mood subscale = 0.330, 95% CI = 0.292–0.368) and cognition (Hedges G for GI = 0.198, 95% CI = 0.181–0.215).

### Efficacy of Lumosity in the ADHD-SRLD cohorts

The preceding Results sections provided a characterization of the ADHD-SRLD cohorts, including a confirmation of cognitive impairment and participant-reported inattentive symptoms. In this section, we examine the effects of Lumosity on these same participants using the same assessments. The efficacy of Lumosity for reducing their inattentive symptoms and improving cognition is demonstrated by greater positive effects on the assessments with increasing doses of CT.

The efficacy analyses examined how much participants who reported ADHD improved on the NCPT and BAMS-7 from the CT provided by Lumosity. Improvement was measured by the change between the first (baseline) and second occurrence of each assessment. Thus, we examined only participants who had taken at least two NCPTs or two BAMS-7s. To examine how change between assessments depended on Lumosity dose, we compared the amount of change in participants with little or no Lumosity use (25 or less gameplays) between assessments to the amount of change in participants with large amounts (400–2000 gameplays). These doses were based on prior work examining the dose-response relation between Lumosity use and improvement on the NCPT, which found little improvement with 25 or less gameplays and substantial improvement with 400 or more^25^. Comparisons of age, gender, and baseline scores between participants with high and low doses of Lumosity use are shown in Table 2.

**Table 2:** Number of gameplays, demographics, and baseline scores of participants receiving high and low doses of Lumosity

	Low-Dose	High-Dose	^1^p-value
NCPT			
Mean (range) Gameplays	11.8 (0–25)	757.5 (400–1994)	NA
N Participants	616	1330	NA
Mean (sd) Age	45.5 (15.9)	45.5 (15.1)	0.981
Gender (% M/F/Unknown)	43.8/48.5/7.6	38.7/54.6/6.7	0.046^*^
Baseline – GI (points)			
Mean (sd) Raw	100.3 (13.3)	102.0 (14.0)	0.012^*^
^2^Mean (sd) Adjusted	95.4 (11.4)	97.0 (11.9)	0.005^**^
BAMS-7			
Mean (range) Gameplays	8.9 (0–25)	816 (400–1989)	NA
N Participants	79	191	NA
Mean (sd) Age	43.8 (15.4)	44.3 (15.2)	0.795
Gender (% M/F/Unknown)	41.8/54.4/3.8	36.7/60.2/3.1	0.679
Baseline – Attention (points)			
Mean (sd) Raw	2.60 (0.94)	2.57 (0.84)	0.814
^2^Mean (sd) Adjusted	2.69 (0.91)	2.65 (0.83)	0.798
Baseline – Mood (points)			
Mean (sd) Raw	2.63 (1.03)	2.77 (0.91)	0.320
^2^Mean (sd) Adjusted	2.76 (1.03)	2.89 (0.89)	0.314

^1^ Based on Welch’s *t* tests for Age and Baseline and Chi-sq tests for Gender.

^2^Adjusted for differences in age and gender between participants receiving high and low doses of CT.

**p < 0.01, *p < 0.05.

The effects of Lumosity dose between assessments were examined on three different types of measure: 1) change in points, 2) effect size of point change, and 3) change in the odds of a clinically meaningful response. To control for demographic differences between participants with high and low doses, we regressed the change scores for each measure on the NCPT and BAMS-7 against age (both linear and quadratic terms) and gender, using all participants who took the NCPT or BAMS-7 twice. Efficacy analyses were performed on the residuals of those participants in the ADHD-SRLD cohorts. Differences in baseline scores between participants with high and low doses of Lumosity (Table 2) had little influence on the results of these analyses, as is shown in Supplementary Note 4. In Supplementary Note 5, the results are shown to remain the same also when participants with intermediate doses are included in the analyses.

#### Change in points following Lumosity use

**Fig. 3:**
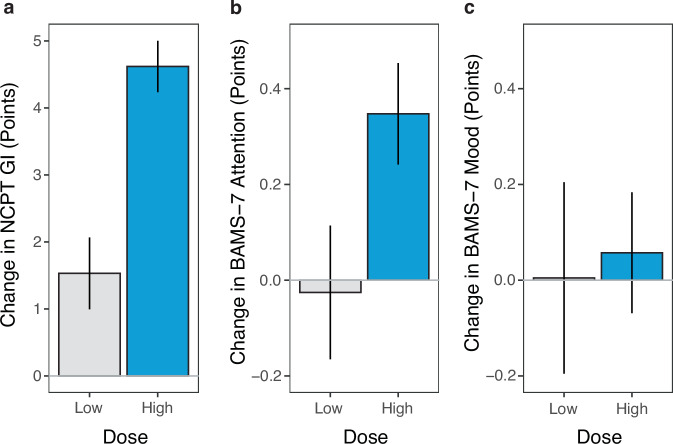
Change on NCPT and BAMS-7 aggregate measures after low and high doses of Lumosity for the ADHD-SRLD cohort. **a** NCPT; **b** BAMS-7 Attention subscale; **c** BAMS-7 Mood subscale. Change is from the baseline to 2nd assessment, and Lumosity dose refers to the amount of intervening Lumosity use. Bars show 95% confidence intervals.

Figure 3 shows change from baseline to the 2nd assessment of the NCPT and BAMS-7 for the three aggregate measures. The effects of dose are illustrated here by separately showing change following little or large amounts of Lumosity use. It can be seen in the figure that a high dose led to a greater increase in points on GI and the Attention subscale, but not on the Mood subscale. Note that following a low dose, there is still an increase in points on GI. This is because performance on the NCPT benefits from repeated testing^25^ regardless of the presence or amount of Lumosity use. In contrast, as is evident in the other low-dose conditions, repeated presentation of the BAMS-7 did not lead to higher ratings. The potential effects of repeated testing are why the following efficacy analyses compare the amount of change between the high- and low-dose conditions (dose effects). The two conditions differ solely in the amount of gameplay between repeated administrations of an assessment.

**Fig. 4:**
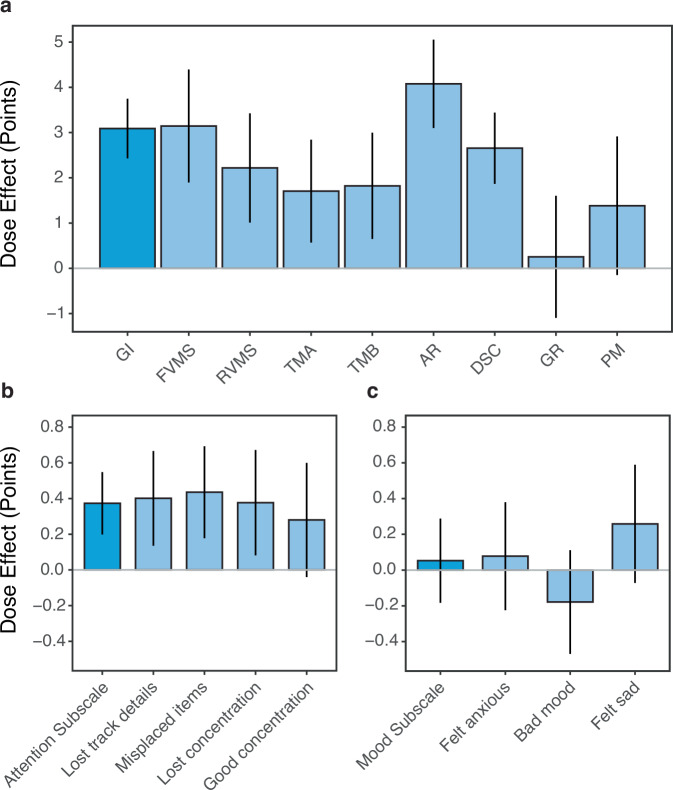
Effects of Lumosity dose (points) on NCPT and BAMS-7 measures for the ADHD-SRLD cohort. **a** NCPT; **b** BAMS-7 Attention subscale; **c** BAMS-7 Mood subscale. The dose effects correspond to differences in change on each measure between participants who engaged in low vs. high amounts of Lumosity use. Bars show 95% confidence intervals. NCPT Measures: GI Grand Index; FVMS Forward Visual Memory Span; RVMS Reverse Visual Memory Span; TMA Trail Making A; TMB Trail Making B; AR Arithmetic Reasoning; DSC Digit-Symbol Coding; GR Grammatical Reasoning; PM Progressive Matrices.

The complete set of dose effects for the NCPT and BAMS-7 are shown in Fig. 4. Here, the difference in amount of change between the high- and low-dose conditions is shown for each subtest, item, and aggregate measure. These differences were tested statistically using Welch’s t-tests, with Bonferroni corrections applied to multiple familywise comparisons involving the eight subtests, four attention items, and three mood items. As implied by Fig. 3, the dose effect is significantly greater than zero for GI (t(1254.1) = 9.192, *p* < 0.0001) and Attention (t(172.1) = 4.221, *p* < 0.0001), but not for Mood (t(144.4) = 0.441, *p* = 0.660). The effects of dose on most of the individual NCPT subtests and BAMS-7 items were consistent with those found on the aggregate measures: With the exception of Grammatical Reasoning (t(1177.2) = 0.369, *p* > 0.1) and Progressive Matrices (t(1206.6) = 1.770, *p* > 0.1), a positive effect was found on all the individual subtests (ts > 2.945, dfs > 1177.4, *p* < 0.03 on the remaining six); With the exception of Good Concentration (t(151.9) = 1.727, *p* > 0.1), a positive effect was found on all the attention items (ts > 2.521, dfs > 153.2, *p* < 0.05 on the remaining three); No significant dose effects were found on the mood items (ts < |1.547 | , dfs < 161.3, *p* > 0.1 on all three).

#### Size of Lumosity dose effects

**Fig. 5:**
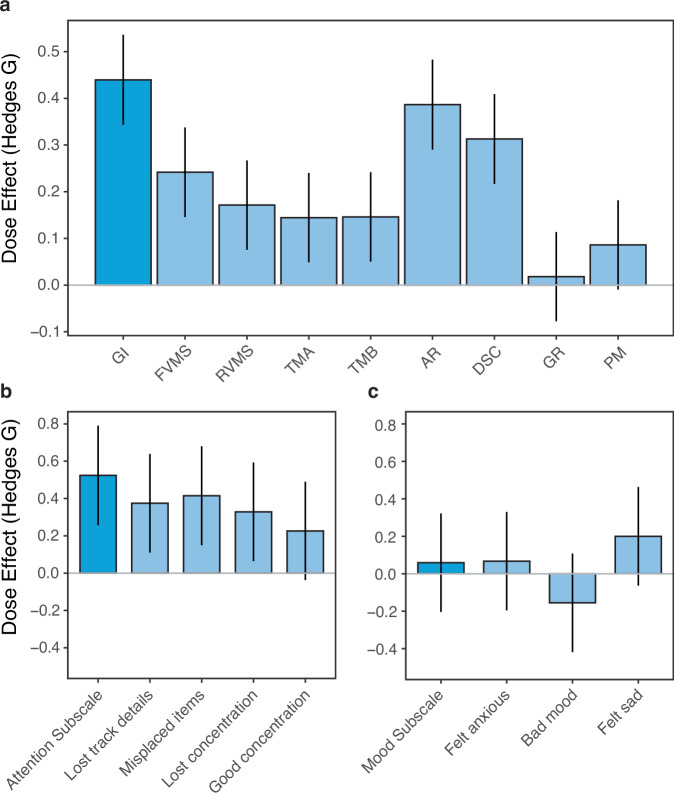
Effect sizes (Hedges G) of Lumosity dose on NCPT and BAMS-7 measures for the ADHD-SRLD cohort. **a** NCPT; **b** BAMS-7 Attention subscale; **c** BAMS-7 Mood subscale. Effect sizes are of differences in change on each measure between participants who engaged in low vs. high amounts of Lumosity (Fig. 4). Bars show 95% confidence intervals. NCPT Measures: GI Grand Index; FVMS Forward Visual Memory Span; RVMS Reverse Visual Memory Span; TMA Trail Making A; TMB Trail Making B; AR Arithmetic Reasoning; DSC Digit-Symbol Coding; GR Grammatical Reasoning; PM Progressive Matrices.

Figure 5 shows the results from Fig. 4 transformed into effect sizes. The Hedges G effect size for each measure is the difference in change between its high- and low-dose conditions (Fig. 4) divided by their pooled standard deviation. They provide an idea of the absolute size of the dose effect on each measure. For example, those on both the GI (0.440, 95% CI = 0.343–0.536) and Attention subscale (0.524, 95% CI = 0.257–0.790) correspond to medium-sized effects. As would be expected, the pattern of results for the effect sizes mirrors that for the unstandardized changes in points (Fig. 4): the effect sizes are positive on most of the NCPT and BAMS-7 attention measures, while change on the BAMS-7 mood measures were uninfluenced by dose (confidence intervals include zero).

#### Dose effects on the odds of a clinically meaningful response

Though our study did not provide participants with clinical care, we reasoned that sufficiently large changes on our outcome measures would suggest the presence of clinically meaningful responses. We therefore examined how Lumosity dose affected the odds of an individual participant exceeding high thresholds of change on the NCPT and BAMS-7. Thresholds for a clinically meaningful response were defined using a distributional approach similar to one used to evaluate whether individual outcomes are clinically significant^42^. A participant in the ADHD-SRLD cohort was classified as a responder on an assessment measure if they achieved a change from baseline of at least 1/2 SD (i.e., Hedges G of 0.5)^42^ of the baseline scores for the entire ADHD-SRLD cohort on that measure. A separate threshold was calculated for each assessment measure and applied to the change scores of the ADHD-SRLD cohort following both low and high doses of Lumosity.

The odds of exceeding the threshold were significantly greater than zero for both assessments at both doses, but greater following high doses (NCPT GI = 0.773, 95% CI = 0.693–0.863; BAMS-7 Attention subscale = 0.854, 95% CI = 0.635–1.147; BAMS-7 Mood subscale = 0.469, 95% CI = 0.340–0.641) than low doses (NCPT GI = 0.342, 95% CI = 0.284–0.411; BAMS-7 Attention subscale = 0.295, 95% CI = 0.164–0.506; BAMS-7 Mood subscale = 0.317, 95% CI = 0.178–0.538). In principle several factors could contribute towards an individual exceeding the threshold, including random variability in the amount of change from baseline, improved scores due to repeated testing, and/or the beneficial effects of CT. To evaluate the unique contribution of CT, we therefore compared the odds between doses.

**Fig. 6:**
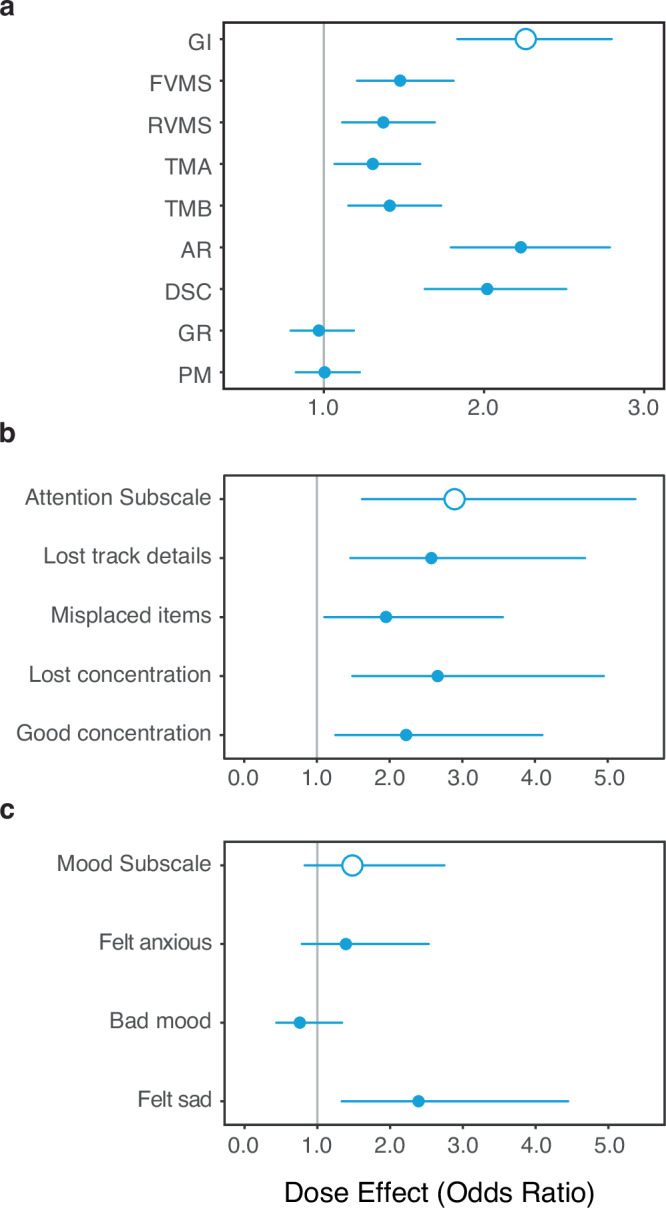
Lumosity dose effects on the odds of a clinically meaningful response on NCPT and BAMS-7 measures for the ADHD-SRLD cohort. **a** NCPT; **b** BAMS-7 Attention subscale; **c** BAMS-7 Mood subscale. A clinically meaningful response on each measure was defined as improvement by at least ½ SD of the baseline scores for the entire ADHD-SRLD cohort on that measure. The Lumosity dose effect is the amount by which the additional Lumosity use in the high dose condition multiplied the odds of a clinically meaningful response in the low dose condition. Bars show 95% confidence intervals. NCPT Measures: GI Grand Index; FVMS Forward Visual Memory Span; RVMS Reverse Visual Memory Span; TMA Trail Making A; TMB Trail Making B; AR Arithmetic Reasoning; DSC Digit-Symbol Coding; GR Grammatical Reasoning; PM Progressive Matrices.

The odds of exceeding the threshold were compared between the two doses by means of a logistic regression analysis. The effects of Lumosity dose on response odds are shown for all NCPT and BAMS-7 measures in Fig. 6. Individual measures are shown along the *y*-axes. The *x*-axes show the relation between the odds of a response in the high and low Lumosity dose conditions. These odds ratios indicate by how much the odds of a response in the low dose condition are multiplied by the additional Lumosity use present in the high dose condition (null effect = 1). As can be seen, the results are similar to those for points and effect sizes. The 95% confidence intervals for GI and six of the subtests (all but GR and PM) exceed 1. This is also the case for the Attention subscale and its component items on the BAMS-7. In contrast, the CIs for the Mood subscale and 2 of its 3 component items include 1.

## Discussion

In this report, we present RWE that Lumosity, a remote digital health technology designed to deliver CT to healthy adults, can improve cognition and reduce inattentive symptoms in a large group of users who reported having received a lifetime diagnosis of ADHD. Over the course of Lumosity training, users were assessed repeatedly with an online neuropsychological test battery (NCPT) and a survey of attention and mood in real-world contexts (BAMS-7). These measures were examined for the ADHD-SRLD cohort, as well as a comparator group of healthy controls.

The primary findings of this study concern the relation between Lumosity use in a real-world setting and the improvements on measures sensitive to ADHD symptoms. The same pattern was found in each of three sets of analyses, involving respectively 1) raw scores, 2) effect sizes, and 3) the odds of a clinically meaningful response. More Lumosity training between successive assessments led to greater positive changes on the NCPT GI and attentional subscale of the BAMS-7. Greater improvements with increasing usage were found for six of the eight NCPT subtests and three of the four BAMS-7 attentional items.

The interpretation of these findings depends both on the clinical status of our ADHD-SRLD cohort and on the sensitivity of our assessments to symptoms of ADHD. Our aim was to examine cognitive improvements in a cohort resembling the population of adults with ADHD found in the real world. Towards this end, we selected members of this cohort from Lumosity users and relied on self-reports of a lifetime medical diagnosis of ADHD. The demographic composition of the resulting cohort, as well as the odds of reporting common psychiatric comorbidities of ADHD, were found to be consistent with those for the general population of adults with ADHD. Our assessments were also found to be sensitive to ADHD symptoms. The ADHD-SRLD cohort showed baseline deficits relative to healthy controls on all NCPT subtests and BAMS-7 items.

Improvements on the assessment measures with more Lumosity training can be viewed in terms of transfer of learning, i.e., the effect of practicing an activity on the performance of a different activity^25,43^. It is important to note in this regard that, though they involve brief computerized cognitive tasks, the NCPT subtests (described individually in the Methods and Supplementary Note 2) are not identical to any of the Lumosity games (described individually in Supplementary Note 6). For example, there is no Lumosity game that involves memorizing reverse sequences of any objects or locations yet there’s an NCPT subtest measuring reverse memory span. The BAMS-7 Attention items concern real-world activities which are even less similar than the NCPT to playing Lumosity games.

For CT to transfer to the performance of any other activity, whether the other activity contributes to neuropsychological test performance or is evaluated on rating scales, the CT and the other activity must involve at least some cognitive processes in common. This transfer can be near or far, in the sense of the differing contexts in which the common cognitive processes are exercised. The borders between near and far transfer are debated, and there’s no clean dividing line^44,45^. But arguably, the NCPT subtests involve a nearer transfer than the BAMS-7 Attention subscale. Interestingly, the affective states rated in the BAMS-7 Mood scale (felt sad, anxious, or in a bad mood over the past week) are not closely linked to specific activities. Perhaps this is why, in contrast to the Attention subscale, there was little or no transfer from Lumosity training to the Mood subscale.

Importantly, the present study provides RWE for transfer. As mentioned in the Introduction, there has been increasing interest in RWE obtained outside of traditional clinical trials^1^. The evidence provided in this report constitutes RWE because it is based on RWD^1^. The study data can be considered RWD in at least two senses. First, it was obtained from a cohort in which ADHD was sometimes accompanied by comorbidities, as is often the case in the real world^7,8,41^. For this reason, it is especially relevant to the potential clinical significance of Lumosity training. Second, the treatment here was non-interventional^46^ in the sense that the amount and timing of Lumosity training were at the users’ discretion. This bears on the efficacy of its remote method of delivery.

Besides providing RWE on Lumosity, by extending prior work, the present study bears on the general use of CT-based digital therapeutics to treat ADHD. The present study involved a large adult cohort; this is important because adult ADHD has received less study than pediatric ADHD, especially with regard to digital health technologies, CT, and RWE. Effective treatment may differ between adult and pediatric populations, given differences between their respective symptoms, brain plasticity and development, and factors influencing compliance and engagement.

Also important is the type of CT delivered by Lumosity. While the Akili therapeutic involves a single game that intensively targets attentional processes, Lumosity includes many different games (Methods and Supplementary Note 6), which, in combination, target multiple cognitive domains. The domains include both executive functions other than attention (e.g., working memory and planning) and non-executive functions (e.g., timing, reasoning). The range of CT and relative emphasis placed on different functions is germane because of the heterogeneity of ADHD symptoms^47^ and the possible involvement of non-executive functions^48^.

The present study also touches on a number of issues relevant to the collection of RWE on digital health technologies more broadly. Examination of dose-response relations provided evidence on the efficacy of Lumosity in the absence of a control group receiving alternative treatment, which is often the case under real-world conditions. The positive relation observed here between the amount of training and improvement on the assessments also highlights the impact of user engagement, which has been identified as a pervasive problem in the remote use of digital health technologies^49^.

Another issue concerns the type of assessment. Remote digital health technologies have tended to rely mainly on self-report questionnaires, in part because such questionnaires are easily digitized and administered. The present study demonstrates the feasibility on a large scale of including also a brief performance-based neuropsychological test battery. Inclusion of both performance-based and self-report measures is important because they are often poorly correlated, which has been attributed in part to their assessing different states and functional abilities^50^.

Interpretation of the present findings is limited by some features of the study. First, the ADHD-SRLD and Healthy Control cohorts were determined by self-report on a survey, rather than clinical diagnosis by physicians. Specifically, participants were asked if they had ever been diagnosed with each of a list of health conditions (Supplementary Table 2). It is therefore possible that a) some of the participants in the ADHD-SRLD cohort never received a traditional clinical diagnosis of ADHD and b) some of the Healthy Controls did. The question in our survey concerning ADHD is, however, similar to that used by the CDC to assess the prevalence of adult ADHD^31^. And though merely a proxy for a clinical diagnosis, it did nonetheless yield an ADHD-SRLD cohort with the expected profile of psychiatric comorbidities and demographic composition.

Related to the diagnosis of ADHD is information concerning possible pharmacological treatment. We have no information concerning whether participants were receiving pharmacological treatment during CT or at the time of assessment. It therefore cannot be determined if or how such treatments affected a) the assessment measures or b) the changes on these measures associated with Lumosity training.

A number of limitations concern the outcome measures themselves. Concordance of the NCPT with measures typically used to assess ADHD is not yet established. Nonetheless, the NCPT subtests have been found to have concurrent validity with the accepted paper and pencil versions on which they are based^28^. Moreover, the deficits found in the ADHD-SRLD cohort relative to the healthy controls in the present study provide evidence that the NCPT is sensitive to ADHD. Another limitation concerns the novelty of the BAMS-7, which may perhaps reduce confidence in its reliability and validity. Note, however, the similarity between items on the Attention subscale and criteria for the inattentive subtype of ADHD in the DSM-5, as well as their similarity to and concordance with items on attention in more established scales (Supplementary Table 4). Measurements in the present study on both BAMS-7 subscales were also found to have acceptable internal consistency and reliability (Methods).

Finally, a causal relation between training with Lumosity and improvements on assessments of ADHD cannot be definitively established in an observational study designed to collect RWE like the present one. For example, it is possible that the greater improvements on the assessments associated with more Lumosity training were due to differences between participants who chose to engage in different amounts of training (e.g., in motivation, medication, or perceived benefits). It is also possible that increased amounts of Lumosity training resulted in greater “placebo effects” on the assessments. But for either of these posited effects to account for the observed pattern of results they would have had to have been selective, influencing only performance on some of the subtests of the NCPT and only responses on the Attention subscale of the BAMS-7.

In sum, despite the above limitations, the present study found evidence of cognitive and attentional benefits in a real-world cohort of adults who reported having received a lifetime diagnosis of ADHD from training with Lumosity under real-world conditions. Moreover, these findings are consistent with those from a recent randomized, double-blind, sham-controlled clinical trial (NCT05296473, pre-registered on ClinicalTrials.gov) involving a digital treatment (Prismira) using Lumosity games in which these limitations were addressed. This latter study involved a cohort of adults with a confirmed and current diagnosis of inattentive or combined-type ADHD. The outcome measures included widely used tests for the assessment of inattention in ADHD. On the basis of positive results from this study, the United States Food and Drug Administration (FDA) recently granted clearance to market Prismira for improving attention function in adult ADHD (Supplementary Note 7). The present study extends the findings from this clinical trial by providing RWE that casual training under naturalistic conditions with a digital health technology designed for use by the general public may improve attention in adult ADHD.

## Supplementary information

Supplementary information

## Data Availability

All data have been made publicly available on the Open Science Framework and can be accessed at https://osf.io/u6dyg/?view_only=35f6b3558e884a5784675ef84f446e15.

## Code availability

No custom code was used in this study.
