# Supplementary material for: Real-world effectiveness of a widely available digital health program in adults reporting a lifetime diagnosis of ADHD
Source: Npj Ment Health Res. 2025 Aug 22;4:38. doi: 10.1038/s44184-025-00157-3 (PMC12373820; doi:10.1038/s44184-025-00157-3)
Supplement: Supplementary file 1 — Supplementary information [file 44184_2025_157_MOESM1_ESM.pdf]

Real-World Effectiveness of a Widely Available Digital Health Program in Adults Reporting a Lifetime Diagnosis of ADHD

Supplementary Materials

Supplementary Note 1: Cohort Definitions and Health Conditions 2

Supplementary Note 2: Neurocognitive Performance Test Subtests Used in the Study 4

Supplementary Note 3: Brief Attention and Mood Survey 9

Supplementary Note 4: Modulation of Dose Effects by Baseline 12

Supplementary Note 5: Dose Effects for Entire ADHD-SRLD cohort 15

Supplementary Note 6: Lumosity Games Used in the Study 18

Supplementary Note 7: FDA Clearance to Market a Digital Therapeutic Using Lumosity Games 32

Supplementary Note 1: Cohort Definitions and Health Conditions

Table 1 in the main text shows the size and demographic composition of the participant cohorts in this study. Here we provide further information about the criteria used to assign participants to these cohorts. Participants were arranged into cohorts in several alternative ways, depending on which analyses were performed. Altogether, eight cohorts were employed in three general classes of analysis (Overall, Baseline, and Efficacy). Each cohort was defined by a combination of two types of criteria: 1) number and type of assessments taken, and 2) health conditions reported on the questionnaire. These two types of criteria are described respectively in Supplementary Tables 1 and 2. When combined, the classification schemes in the two tables yield the two cohorts used in the Overall Analyses, four cohorts used in the Baseline Analyses, and two cohorts used in the Efficacy Analyses. These are the cohorts listed in Table 1 in the main text and shown here in Supplementary Table 3.

Supplementary Table 1. Inclusion criteria involving assessments used to define study cohorts.

	Cohorts				
	Overall	Baseline		Efficacy	
**Assessments**	**NCPT or BAMS-7**	**NCPT**	**BAMS-7**	**NCPT**	**BAMS-7**
**At least 1 NCPT**	Include	Include	---	---	---
**At least 1 BAMS-7**	Include	---	Include	---	---
**At least 2 NCPTs**	---	---	---	Include	---
**At least 2 BAMS-7s**	---	----	---	---	Include

Note. Some participants who took the NCPT did not take the BAMS-7 and vice versa. The Overall cohorts included participants who took either (or both) the NCPT or BAMS-7 at least once. The Baseline NCPT cohorts included only participants who took the NCPT at least once, and the Efficacy NCPT cohorts included only participants who took the NCPT at least twice. Likewise, the Baseline BAMS-7 and Efficacy BAMS-7 cohorts included respectively only those participants who took the BAMS-7 at least once and at least twice.

Supplementary Table 2. Exclusion and inclusion criteria involving health conditions used to define study cohorts.

	Cohort				
	Overall		Baseline		Efficacy
**Health Condition**	**ADHD -**	**ADHD+**	**Healthy Controls**	**ADHD+**	**ADHD+**
**Alzheimer's Disease**	Exclude	Exclude	Exclude	Exclude	Exclude
**Dementia**	Exclude	Exclude	Exclude	Exclude	Exclude
**Huntington's Disease**	Exclude	Exclude	Exclude	Exclude	Exclude
**Mild Cognitive Impairment**	Exclude	Exclude	Exclude	Exclude	Exclude
**Multiple Sclerosis**	Exclude	Exclude	Exclude	Exclude	Exclude
**Parkinson's Disease**	Exclude	Exclude	Exclude	Exclude	Exclude
**Stroke**	Exclude	Exclude	Exclude	Exclude	Exclude
**Traumatic Brain Injury**	Exclude	Exclude	Exclude	Exclude	Exclude

**ADHD**	Exclude	Include	Exclude	Include	Include
**Anxiety**	---	---	Exclude	---	---
**Autism**	---	---	Exclude	---	---
**Cancer- Breast**	---	---	Exclude	---	---
**Cancer-Chemofog**	---	---	Exclude	---	---
**Cancer-Colon**	---	---	Exclude	---	---
**Cancer-Kidney**	---	---	Exclude	---	---
**Cancer-Lung**	---	---	Exclude	---	---
**Cancer-Ovarian**	---	---	Exclude	---	---
**Cancer-Prostate**	---	---	Exclude	---	---
**Cancer-Thyroid**	---	---	Exclude	---	---
**Cystic Fibrosis**	---	---	Exclude	---	---
**Depression**	---	---	Exclude	---	---
**Diabetes**	---	---	Exclude	---	---
**Dyslexia**	---	---	Exclude	---	---
**Heart Disease**	---	---	Exclude	---	---
**Hyper- or Hypo-Thyroidism**	---	---	Exclude	---	---
**Learning Disability**	---	---	Exclude	---	---
**Muscular Dystrophy**	---	---	Exclude	---	---
**Post-Traumatic Stress Disorder**	---	---	Exclude	---	---
**Schizophrenia**	---	---	Exclude	---	---
**Seizures**	---	---	Exclude	---	---
**Sleep Disorder**	---	---	Exclude	---	---
**Substance Abuse Disorder**	---	---	Exclude	---	---

Note. Participants who reported any of the eight neurological conditions reported at the top of the Health Condition list (leftmost column) were excluded from all cohorts. The ADHD+ cohorts included only participants who reported ADHD and the ADHD- cohorts included only participants who didn't. The Healthy Control cohorts included only participants who did not report any of the health conditions.

Supplementary Table 3. Health conditions of and assessments taken by cohorts employed in each analysis type.

Overall Analyses (Results Section 1)
1) ADHD- / >= 1 NCPT and/or 1 BAMS-7
2) ADHD+ / >= 1 NCPT and/or 1 BAMS-7

Baseline Analyses (Results Section 2)
3) ADHD+ / >=1 NCPT
4) Healthy Controls / >= 1 NCPT
5) ADHD+ / >= 1 BAMS-7
6) Healthy Controls / >= BAMS-7

Efficacy Analyses (Results Section 3)
7) ADHD+ / >= 2 NCPTs
8) ADHD+ / >=2 BAMS-7s

Supplementary Note 2: Neurocognitive Performance Test (NCPT) Subtests Used in the Study

NCPT Conceptual Framework

The subtests of the NCPT are based on or inspired by existing neuropsychological assessments. Each subtest has an interactive, clear tutorial prior to assessment administration to ensure a minimum level of proficiency with the task. Each assessment also has dynamically-generated content, which allows for increased number of trials and repeatability of the subtest. These assessments feature a white background with black text and fill a window on the computer screen 640 pixels in width by 480 pixels in height. The NCPT subtests used in the study are described below.

Arithmetic Reasoning

Arithmetic Reasoning is a cognitive task in which individuals solve basic arithmetic questions, written in words. Arithmetic Reasoning is similar to sections of assessments of mathematical processing that present problems in modalities other than Arabic numerals [1].

This assessment requires the participant to rapidly and accurately solve simple arithmetic problems that are written in words – for example, "Four plus two =". The answer is input using the number keys on the keyboard ("6" in the example above). For addition and multiplication problems, operands are uniformly sampled from the integers in the range 1-9, and for subtraction and division problems, the second operand and solution are uniformly sampled from the integers in the range 1-9. The addition operator is used in the first five trials. Subsequent trials use addition 50% of the time. In non-addition trials, operators are chosen uniformly from subtraction, multiplication, and division. The assessment lasts 90 seconds, and the total number of correct responses in that time period is the dependent measure. It is considered a measure of problem solving ability.

Arithmetic Reasoning

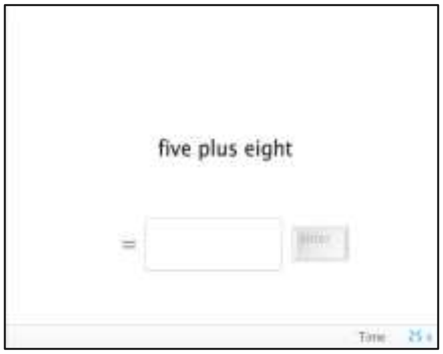

Digit Symbol Coding

Digit Symbol Coding is based on the Digit Symbol Substitution Task in the Brief Assessment of Cognition in Schizophrenia (BACS) and is used to measure information processing speed, the principal components of which are visual search and memory [2].

A legend with numbers 1 to 9 and corresponding abstract symbols is presented across the top of the screen. The height of the numbers is equal to 1/24 the height of the screen, and symbols fill a space with height and width equal to 1/12 the height of the screen. The symbols are randomly selected from a pool of 18 possible symbols. In the middle of the screen a large, two-section box (top and bottom sections each having a height equal to ¼ of the screen height) is presented with the symbol in the top section. The participant is required to use the number keyboard to type the number that corresponds to the symbol. Once a number is pressed, it moves on to the next trial. Participants can see the next symbol grayed out, but they cannot go back to a previous trial. The assessment is timed for 90 seconds and the dependent measure is the total number of correct trials minus incorrect trials.

Digit Symbol Coding

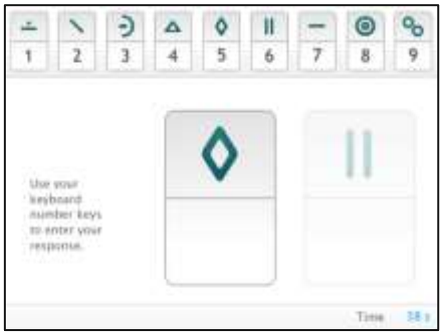

Forward and Reverse Visual Memory Span

Memory span is a common measure of visual short-term memory and functionally appears to measure the number of discrete units over which an individual can successively distribute attention and still organize into a working unit. To generalize, forward memory span refers to the ability of an individual to reproduce immediately, after one presentation, a series of discrete stimuli in their original order whereas reverse memory span requires reproducing the order in reverse.

The Visual Memory Span tasks are derived from the Corsi block-tapping test, which is a psychological test that assesses visuospatial short term working memory. The Corsi block-tapping task involves mimicking a researcher as he/she taps a sequence of up to nine identical spatially separated blocks. The backward Corsi block-tapping task is a slightly altered version of the original Corsi block-tapping task, and is considered a measure of working memory. In the backward task, the subjects are asked to watch the sequence and instead of mimicking the researcher's pattern, they are asked to repeat the sequence in reverse order. In computerized versions of the Visual Memory Span tasks, instead of cubes to be tapped on a board, the tests consist of circles that flash on a computer screen and participants reproduce the sequences by using a mouse to click on the circles [3, 4].

Blue circles with radii equal to 1/20 of the window height are placed at randomized, non-overlapping spatial locations and individually highlighted in orange following a particular sequence. Circles are highlighted for 500 msec with a 100 msec inter-stimulus interval. The participant is asked to recall the sequence by clicking on each circle in the same order as originally presented. The length of the sequence increases by one every three trials. The session ends when the participant gives three incorrect answers at the same span level. The total number of correct responses is the dependent measure.

Forward Memory Span

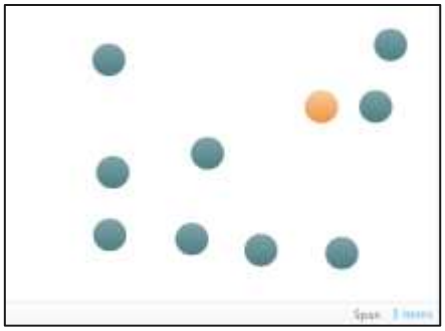

Reverse Memory Span

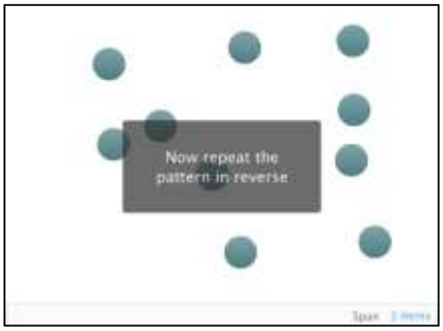

Trail Making A and B

The Trail Making Test (TMT) is one of the most popular neuropsychological tests and is included in most test batteries [5, 6]. The TMT provides information on attention and speed of processing. Originally, it was part of the Army Individual Test Battery [7] and subsequently was incorporated into the Halstead–Reitan Battery [8].

In Trail Making A, blue circles numbered from 1 to 24 are arranged in 6 possible layouts with non-overlapping spatial locations. Layouts are normalized for completion time with total path length equal to 5 1/3 times the screen height, as well as equivalent circle-to-circle distance and angle distributions and circle density distributions. The circles have radii equal to 1/25 of the screen height and the number height is equal to 1/24 of the screen height. The participant is required to click on the circles sequentially connecting the encircled numbers. When clicked the blue circles change color to orange and a straight line appears connecting the circles. Task requirements are similar for Trail Making B except the circles now include numbers (1 to 12) and capital letters (A to L) and the participant must alternate between numbers and letters (e.g., 1, A, 2, B, 3, C, etc.). The timer begins when the participant clicks number 1. If the participant clicks on an incorrect circle, an X appears and they must go back to the previous circle. The amount of time required to complete the task is the dependent measure.

Trail Making A

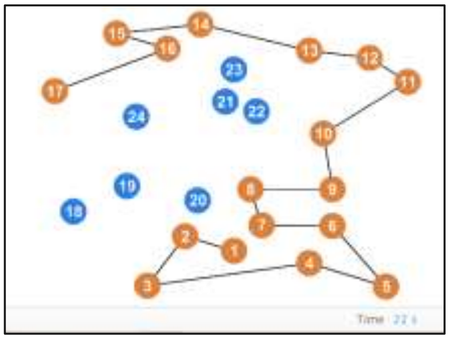

Trail Making B

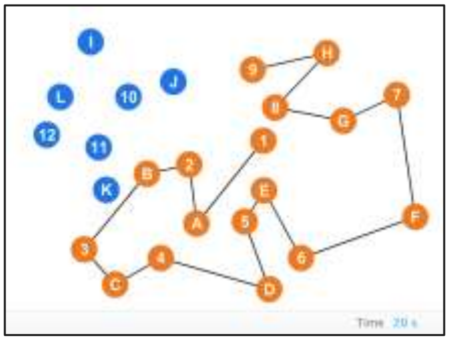

Grammatical Reasoning

The grammatical reasoning test is a test of the ability to carry out mental operations involving chains of logical reasoning. The test is derived from Baddeley's Grammatical Reasoning Test [9] and measures

the participant's facility to rapidly and accurately evaluate a potentially confusing grammatical statement.

A blue square and a blue equilateral triangle, each with height equal to 1/5 of the window height are shown side by side, with a logical statement written below. For example, the square may be positioned to the left of the triangle on a particular trial. The participant could be prompted with a statement of the form, "The square is not to the left of the triangle." In this case, the answer would be "false." The participant responds whether the statement is true or false by pressing a key on the keyboard that is indicated as corresponding to true or false. The probability that the statement includes a negative ("not") is 50%. The net number of correct responses (number correct – number incorrect) in 45 seconds is the dependent measure, with a floor of zero. This test is a measure of cognitive flexibility and logical reasoning.

Grammatical Reasoning

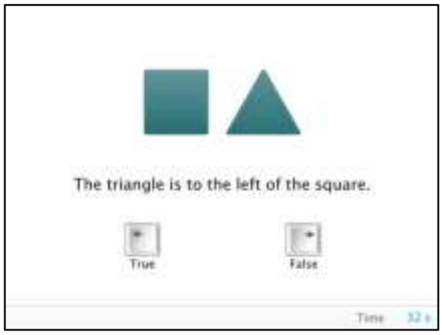

Progressive Matrices

The Progressive Matrices test was inspired by Raven's Progressive Matrices [10], with the exception that the NCPT version has dynamically generated problems. Matrix reasoning assessments require the participant to determine which stimulus most logically completes a multi-dimensional pattern. In this version of matrix reasoning, the participant is shown a 3x3 grid (each grid slot has width and height equal to 1/5 the window height) with abstract stimuli in the 8 upper-left slots. The task is to choose which of six possible answer choices best completes the pattern in the grid. The assessment is made up of 17 problems of increasing difficulty that are algorithmically generated from a set of parameters. The 17 problems are divided into three broad problem types: progression matrix, orbital/lateral movement, and Boolean logic. The first 12 trials involve progression matrix rules of increasing complexity. Characteristics of the stimuli that may change include shape, number, color, rotation angle, and size. These patterns may progress across the matrix horizontally, vertically, from upper-left to lower-right diagonal, or from lower-left to upper-right diagonal. Trials 13-15 involve orbital or lateral movement in which square grids or circular orbits are partially filled with elements that progress according to a lateral or rotational movement rule. Trials 16-17 involve Boolean logic in which spatial patterns are combined using Boolean operators such as AND, OR, and XOR. For each problem type, the correct answer is indicated regardless of whether the participant answers correctly. The assessment ends once the participant completes 17 trials or answers three consecutive trials incorrectly. The total number correct is the dependent measure. Matrix reasoning is considered a measure of problem solving and fluid reasoning (often referred to as fluid intelligence).

Progressive Matrices

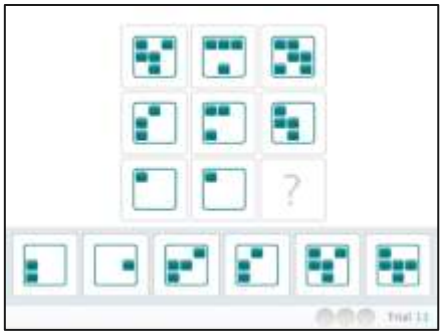

References

1. Deloche G, Seron X, Larroque C, Magnien C, Metz-Lutz MN, Noel MN, et al. Calculation and number processing: assessment battery; role of demographic factors. J Clin Exp Neuropsychol. 1994;16(2):195-208. doi: 10.1080/01688639408402631

2. Royer FL. Spatial orientational and figural information in free recall of visual figures. Journal of Experimental Psychology. 1971;91(2):326. doi.org: 10.1037/h0031846

3. Schoenberg MR, Scott JG. The little black book of neuropsychology: A syndrome-based approach. New York: Springer; 2011. doi.org: 10.1007/978-0-387-76978-3

4. Pearson D, Sahraie A. Oculomotor control and the maintenance of spatially and temporally distributed events in visuo-spatial working memory. The Quarterly Journal of Experimental Psychology Section A. 2003;56(7):1089-111. doi: 10.1080/02724980343000044

5. Camara WJ, Nathan JS, Puente AE. Psychological test usage: Implications in professional psychology. Professional psychology: Research and practice. 2000 Apr;31(2):141. doi: 10.1037/0735-7028.31.2.141

6. Rabin LA, Barr WB, Burton LA. Assessment practices of clinical neuropsychologists in the United States and Canada: A survey of INS, NAN, and APA Division 40 members. Archives of Clinical Neuropsychology. 2005 Jan 1;20(1):33-65. doi: 10.1016/j.acn.2004.02.005

7. Personnel Research Section, Classification and Replacement Branch, AGO. The new army individual test of general mental ability. Psychol Bull. 1944;41:532-538. doi: 10.1037/h0063394

8. Reitan RM, Wolfson D. The Halstead-Reitan neuropsychological test battery: Theory and clinical interpretation. Tucson, AZ: Neuropsychology Press; 1985.

9. Baddeley AD. A 3 min reasoning test based on grammatical transformation. Psychon Sci. 1968;10(10):341-342. doi:10.3758/BF03331551

10. Penrose LS, Raven JC. A new series of perceptual tests: preliminary communication. British Journal of Medical Psychology. 1936. doi: 10.1111/j.2044-8341.1936.tb00690.x

Supplementary Note 3: Brief Attention and Mood Survey (BAMS-7)

The present study found the ADHD-SRLD cohort to be impaired relative to healthy controls on all items of the BAMS-7. This finding is consistent with the content of these items. As shown in Supplementary Table 4, the wording of the BAMS-7 items resembles that found in the Diagnostic and Statistical Manual of the American Psychiatric Association[1] (DSM-5) and that of items on several well-validated scales.

The items on the BAMS-7 Attention subscale (top 4) resemble criteria for the inattentive subtype of ADHD in the DSM-5. These items also resemble those assessing attention on other scales[2], including the Adult ADHD Self-report Scale[3] (ASRS) and the Attention-Related Cognitive Errors Scale[4] (ARCES).

The items on the BAMS-7 Mood subscale (bottom 3) resemble items on scales sensitive to comorbidities often associated with ADHD.[2] These include the Generalized Anxiety Disorder Assessment[5] (GAD-7), the Patient Health Questionnaire-9[6] (PHQ-9, a measure of depression), and the Positive and Negative Affect Schedule[7](PANAS).

Ratings on the BAMS-7 items were also found to be significantly correlated (all p's < 0.001) with similar items on the other scales. The correlations displayed in the table are based on ratings obtained remotely via MTurk from 150 participants (20-63 years old, mean age = 40.16 yrs, 50% male, 72% bachelor's degree or higher) from the general US population without further restrictions.[2] (Note that many of the correlations are negative because a higher score on the BAMS-7 indicates better attention or mood, while a higher score on some of the other scales indicates more inattention or poorer mood.)

In sum, the information presented in Supplementary Table 4 both confirms the face validity of the BAMS-7 and demonstrates convergent validity with items on other scales of attention and mood. For further details, see [2].

Supplementary Table 4. Comparison of BAMS-7 items with DSM-5 criteria for ADHD and with related items on other scales of attention and mood.

BAMS-7	DSM-5 ADHD	ASRS ADHD	ARCES	PANAS	PHQ-9	GAD-7
**During the last month, how often have you…**		**Over the last 6 months…**		**Over the past few weeks…**	**Over the last few weeks…**	**Over the last few weeks…**
Lost track of details as you were reading and needed to go back and reread sections?	Often fails to pay close attention to details or makes mistakes	-	When reading I find that I have read several paragraphs without being able to recall what I read<br><br>r = -0.623	-	-	-
Misplaced items (e.g., reading glasses, keys)	Often loses things necessary for tasks	How often do you misplace or have difficulty finding things at	I have absent-mindedly misplaced frequently	-	-	-

around the house?	and activities	home or at work?<br><br>*r* = -0.575	used objects, such as keys, pens, glasses, etc.<br><br>*r* = -0.570			
Found yourself losing concentration during a conversation?	Often does not seem to listen when spoken to directly	How often do you have difficulty concentrating on what people say to you, even when they are speaking to you directly?<br><br>*r* = -0.558	I have lost track of a conversation because I zoned out when someone else was talking.<br><br>*r* = -0.676	-	-	-
**Rate your experience over the last week…**						
My ability to concentrate was good.	Often has trouble holding attention on tasks or play activities ; Is often easily distracted	-	-	Attentive<br><br>r = 0.598<br><br>Alert<br><br>r = 0.454	Trouble concentrating on things, such as reading the newspaper or watching television<br><br>*r* = -0.403	-
I felt anxious.	-	-	-	Nervous<br><br>*r* = 0.711	-	Feeling nervous, anxious, or on edge<br><br>*r* = -0.642
I was in a bad mood.	-	-	-	Irritable<br><br>r = -0.578	Feeling bad about yourself - or that you are a failure or have let yourself or your family down<br><br>*r* = -0.470	-
I felt sad for no obvious reason.	-	-	-	Upset<br><br>*r* = -0.627	Feeling down, depressed, or hopeless<br><br>*r* = -0.665	-

Note. All p's < 0.001.

References

1. American Psychiatric Association. (2013). *Diagnostic and statistical manual of mental disorders* (5th ed.). https://doi.org/10.1176/appi.books.9780890425596

2. Madore, KP, Osman, AM, Kerlan, KR, & Schafer, RJ. Reliability and Validity of the Brief Attention and Mood Scale of 7 Items (BAMS-7): A Self-Administered, Online, and Real-World Measure. *MedRxiv.* doi: https://doi.org/10.1101/2024.04.05.24305401

3. Kessler, R. C., Adler, L., Ames, M., Demler, O., Faraone, S., Hiripi, E. V. A., ... & Walters, E. E. (2005). The World Health Organization Adult ADHD Self-Report Scale (ASRS): A short screening scale for use in the general population. *Psychological Medicine*, *35*(2), 245-256. doi: https://doi.org/10.1017/S0033291704002892

4. Cheyne, J. A., Carriere, J. S., & Smilek, D. (2006). Absent-mindedness: Lapses of conscious awareness and everyday cognitive failures. *Consciousness and Cognition*, *15*(3), 578-592. doi: https://doi.org/10.1016/j.concog.2005.11.009

5. Spitzer, R. L., Kroenke, K., Williams, J. B. W., & Löwe, B. (2006). *Generalized Anxiety Disorder 7 (GAD-7)*. APA PsycTests. doi: https://doi.org/10.1001/archinte.166.10.1092

6. Kroenke K, Spitzer R L, Williams J B (2001). The PHQ-9: Validity of a brief depression severity measure. *Journal of General Internal Medicine*, *16*(9): 606-613. doi: https://doi.org/10.1046/j.1525-1497.2001.016009606.x

7. Watson, D., Clark, L. A., & Tellegen, A. (1988). Development and validation of brief measures of positive and negative affect: The PANAS scales. *Journal of Personality and Social Psychology*, *54*(6), 1063. doi: https://doi.org/10.1037/0022-3514.54.6.1063

Supplementary Note 4: Modulation of Dose Effects by Baseline

As mentioned in the main text (Table 2), there were significant baseline differences in NCPT GI between those participants reporting a lifetime diagnosis of ADHD who engaged in negligible amounts of Lumosity training (25 or less gameplays) vs. those who engaged in substantial amounts (400 or more gameplays). Here we consider whether such baseline differences between participants with different dose levels could have contributed to the pattern of dose effects observed on the NCPT GI, BAMS-7 Attention subscale, and BAMS-7 Mood subscale (Figure 4).

Correlation between baseline and change scores. First, it should be noted that baseline (prior to training) scores were negatively correlated with change (post-training minus prior to training) scores on all three measures. The correlations for the entire (all levels of training) group of participants reporting ADHD are: NCPT GI ($r(7{,}072) = -0.248$, $p < 0.001$); BAMS-7 Attention subscale ($r(740) = -0.433$, $p < 0.001$); BAMS-7 Mood subscale ($r(740) = -0.499$, $p < 0.001$). The corresponding three linear functions relating baseline and change scores are displayed in Supplementary Figure 1.

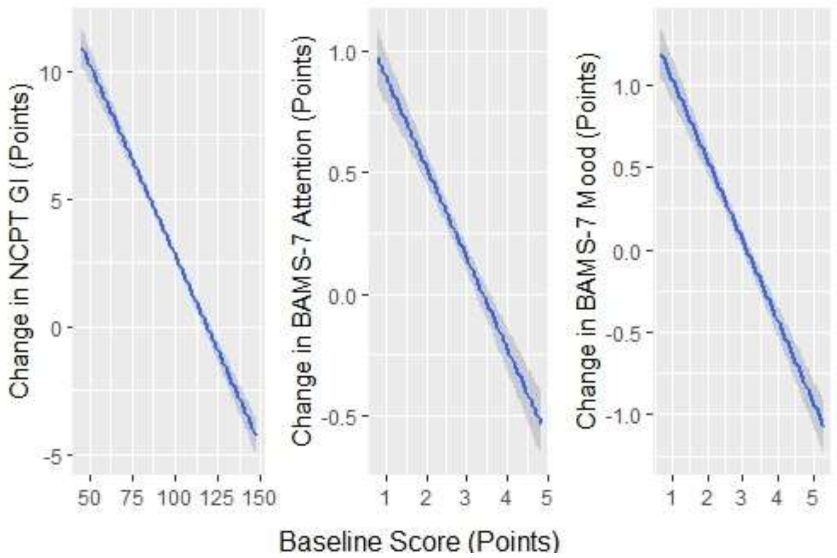

Supplementary Figure 1. Change on NCPT GI and BAMS-7 Attention and Mood subscales as a linear function of baseline score for participants reporting a lifetime diagnosis of ADHD. Change is from the baseline to 2nd assessment. Grey shading shows the 95% confidence intervals around each function.

It can be seen in the figure that the highest baseline scores for each measure are associated with negative change scores. This suggests that the negative correlations are due, at least in part, to regression towards the mean. Note that the mere presence of a regression towards the mean should not affect the expected value of the change scores (estimated by their mean). It remains possible, however, that the negative correlations also reflect systematic effects, e.g., greater effects of training in participants with lower baselines.

Potential influence of baseline on dose effects. Dose effects are defined as the difference in change scores between participants engaging in substantial (≥ 400 gameplays) and negligible (≤ 25 gameplays) amounts of Lumosity training. Because of this subtraction, a systematic effect of baseline on change scores could influence the magnitude, significance, or even direction of dose effects. If there was such a

systematic effect for the NCPT GI, the dose effect on this measure reported in the main text would be an underestimate. Baseline scores for the NCPT GI were found to be significantly higher for participants with more training (Table 2). Thus adjusting change scores to compensate for their inverse relation to baseline would increase the size of the positive dose effect.

A systematic effect of baseline on change scores might also influence the pattern of dose effects observed on the BAMS-7 Attention and Mood subscales. Baseline scores on the Attention subscale were numerically, albeit non-significantly, lower for participants who engaged in more training. Adjusting for baseline could conceivably reduce the magnitude of the positive dose effect on this measure, causing it to become non-significant. Baseline scores on the Mood subscale were numerically but non-significantly higher for participants with more training. Adjusting for baseline might cause the null effect of dose observed on this measure to become significantly positive.

Actual influence of baseline on dose effects. To examine the influence of these potential systematic effects of baseline on the pattern of dose effects, we performed three linear regressions. The dependent variable in each was the age- and gender-corrected change scores for one of the three assessment measures. The independent variables in all regressions were the amount of Lumosity training (Dose), Baseline Score, and the interaction between the two. Of particular interest was whether the effects of Dose reported in the main text (Figure 4) remained significant (in the same direction) or non-significant and whether Dose interacted with Baseline. The results are shown in Supplementary Table 5.

Supplementary Table 5. Linear regressions examining the modulation of dose effects by baseline scores.

**Model: Change_on_Assessment ~ Dose * (Baseline – Mean (Baseline))**				
**Change on NCPT Grand Index**				
**Coefficients**	**Estimate**	**Std. Error**	**t value**	**Pr(>|t|)**
Intercept (Low Dose, Mean BL)	1.350	0.272	4.960	7.68e-07 ***
Dose	3.357	0.329	10.208	< 2e-16 ***
Baseline	-0.167	0.024	-7.042	2.61e-12 ***
Dose * Baseline	-0.008	0.028	-0.279	0.78
**Change on BAMS-7 Attention**				
**Coefficients**	**Estimate**	**Std. Error**	**t value**	**Pr(>|t|)**
Intercept (Low Dose, Mean BL)	-0.020	0.072	-0.278	0.7815
Dose	0.364	0.086	4.248	2.98e-05 ***
Baseline	-0.258	0.080	-3.228	0.0014 **
Dose * Baseline	-0.149	0.097	-1.535	0.1261
**Change on BAMS-7 Mood**				
**Coefficients**	**Estimate**	**Std. Error**	**t value**	**Pr(>|t|)**
Intercept (Low Dose, Mean BL)	-0.036	0.090	-0.399	0.690
Dose	0.110	0.107	1.023	0.307
Baseline	-0.430	0.088	-4.883	1.81e-06 ***
Dose * Baseline	0.009	0.110	0.079	0.937

***p < 0.001, **p < 0.01

The estimates of dose effects for the three measures in the table are close in size to those reported for points in the main text (Figure 4). Similarly, as reported in the main text, the dose effects are significant for GI and the BAMS-7 Attention subscale but not for the BAMS-7 Mood subscale. Consistent with Supplementary Figure 1 and the correlations between baseline and change score reported above, the Baseline estimates for all three measures are negative and significant. Importantly, the interaction between Dose and Baseline is not significant for any of the measures.

Conclusions. No evidence was found that the size of the dose effect on any of the measures depends on baseline score. Adding baseline score to the regression models had no detectable effect on the pattern of dose effects reported in the main text.

Supplementary Note 5: Dose Effects for Entire ADHD-SRLD Cohort

The efficacy findings in the main text (Results section III, Figs 3-6) show how performance on the NCPT and ratings on the BAMS-7 differs between participants in the ADHD-SRLD cohort who engaged in negligible amounts of Lumosity training (25 or less gameplays) and those who engaged in substantial amounts (400 or more gameplays). Here we confirm that the same pattern of dose-response relations applies when participants with intermediate amounts of gameplay are also included in the analyses.

The following analyses included all participants in the ADHD-SRLD cohort with 2000 gameplays or less (98.7% for NCPT and 97.2% for BAMS-7) and estimate continuous functions relating change on the assessments to amount of gameplay. To control for age and gender differences between participants with different amounts of gameplay, assessment scores were adjusted in the same way as in the prior efficacy analyses (Results section III).

Dose Histograms. Supplementary Figure 2 displays the number of participants with different amounts of gameplay between their baseline and 2nd NCPT or BAMS-7. As can be seen, 1) the vast majority of participants had less than 1,000 gameplays and 2) considerably more participants took two NCPTs than took two BAMS-7s (see also Table 1).

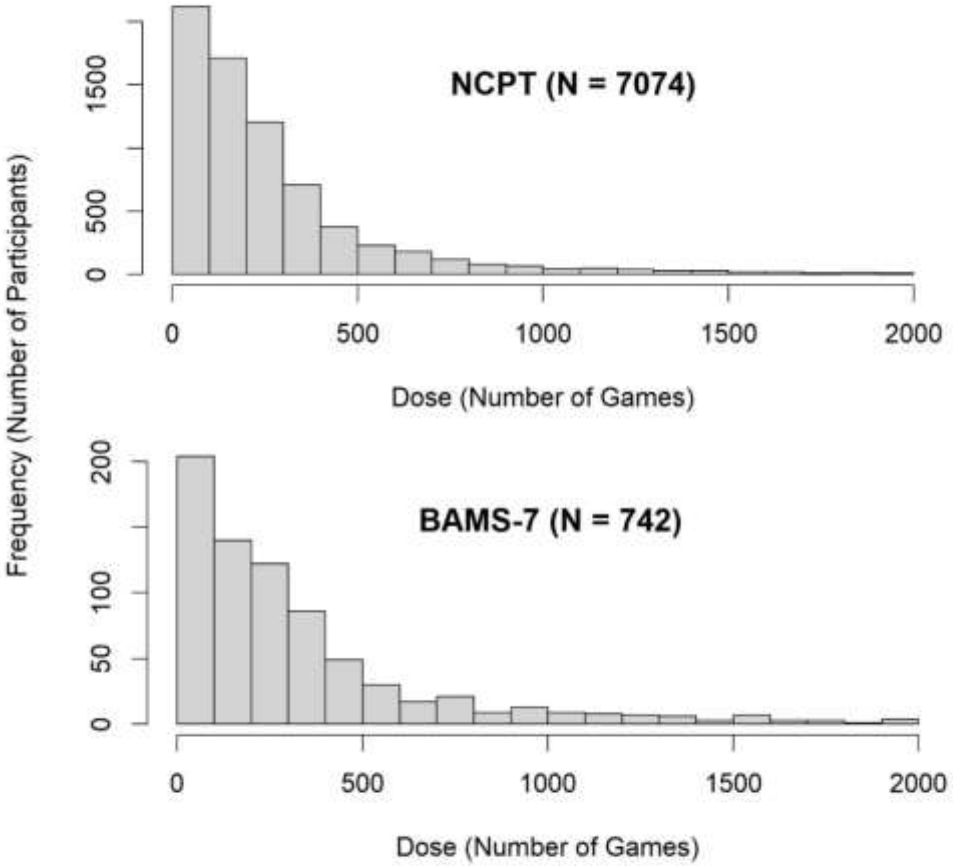

Supplementary Figure 2. Number of participants in the ADHD-SRLD cohort with different amounts of gameplay between their baseline and 2nd NCPT or BAMS-7.

Dose-Response Functions. Supplementary Figure 3 shows continuous dose-response functions for the aggregate measures, i.e., NCPT GI and the Attention and Mood scales on the BAMS-7. The plotted lines are logarithmic functions fit to all 7074 individual data points for the NCPT and 742 data points for the two BAMS-7 scales. The plotted points are the means of deciles for the NCPT (each based on 700+ points) and quintiles for the BAMS-7 scales (each based on almost 150 points). The latter are included just to show that the functions approximately fit the data. But regardless of their precise fit, it can be seen that NCPT GI and BAMS-7 Attention scores increase with more gameplay, but that additional gameplay has diminishing returns. For the BAMS-7 Mood scale, increased gameplay has no apparent effect.

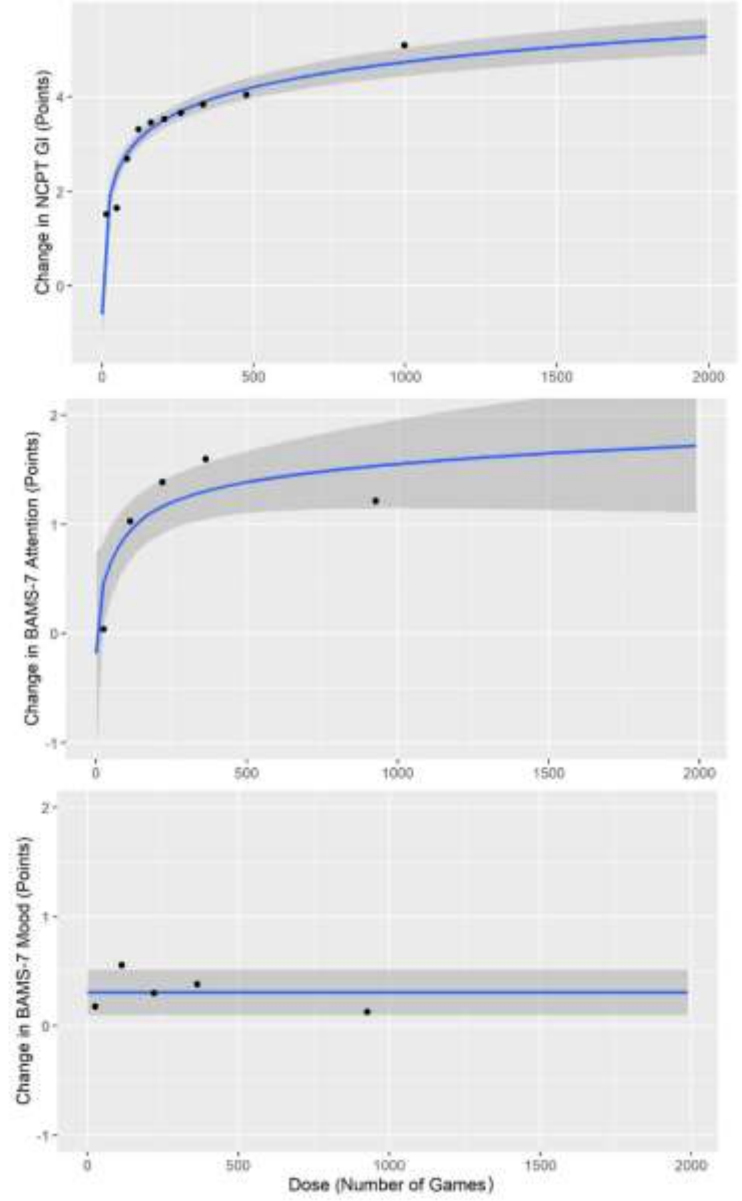

Supplementary Figure 3. Change on NCPT GI and BAMS-7 Attention and Mood subscales in the ADHD-SRLD cohort as a function of number of gameplays between baseline and 2nd assessments. The lines show logarithmic functions fit to all 7074 individual data points for the NCPT and 742 data points for the two BAMS-7 scales. The plotted points are the means of deciles for the NCPT and quintiles for the BAMS-7 scales. Grey shading shows the 95% confidence intervals around each function.

Correlations Between Dose and Assessment Scores. Supplementary Figure 4 shows the relation between amount of gameplay and the scores on all the individual NCPT subtests and BAMS-7 items. Shown here are the correlations (and 95% CIs) between the logarithm of amount of gameplay and the scores on each subtest and item. The correlations are significantly positive for GI and all NCPT subtests except Grammatical Reasoning. Similarly, all are significantly positive for the aggregate and individual items on the BAMS-7 Attention Scale. In contrast, no significant correlations were found between gameplay and any of the BAMS-7 Mood measures.

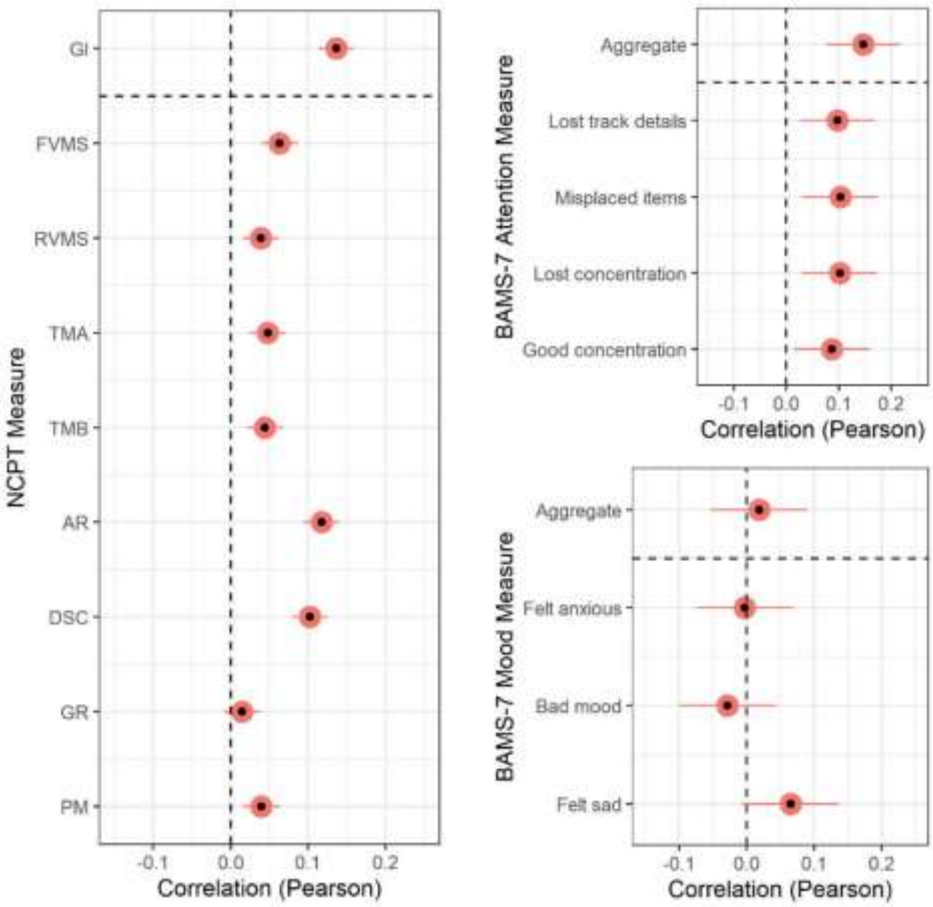

Supplementary Figure 4. Correlations between number of gameplays and assessment scores in the ADHD-SRLD cohort. Pearson correlations are shown for the aggregate measures and individual subtests and items on the NCPT and BAMS-7. Each correlation is between the logarithm of the number of games between the baseline and 2nd assessment and the change in assessment score. Dots show the correlations and error bars show their 95% confidence intervals. NCPT Measures: GI = Grand Index; FVMS = Forward Visual Memory Span; RVMS = Reverse Visual Memory Span; TMA = Trail Making A; TMB = Trail Making B; AR = Arithmetic Reasoning; DSC = Digit-Symbol Coding; GR = Grammatical Reasoning; PM = Progressive Matrices.

Conclusions. The same dose-response relations in the ADHD-SRLD cohort were observed here as in the efficacy analyses presented in the main text. More gameplay was associated with greater change on the NCPT and BAMS-8 Attention subscale, but not the BAMS-7 Mood subscale (compare Supplementary Figure 4 and Figure 4). This pattern was observed 1) for both continuous dose-response functions and comparisons between subgroups of participants with negligible vs. substantial amounts of gameplay and 2) regardless of whether participants with intermediate amounts of gameplay are included.

Supplementary Note 6: Lumosity Games Used in the Study

Cognitive training was provided via the Lumosity program, which included 69 different games across web and mobile apps over the time period in which the study data were collected. (Games were introduced and discontinued over time, so the exact number of available games varied.) Lumosity games are modeled on paradigms used to study specific cognitive functions in the lab or clinic. Based on their primary cognitive demands, they can be organized into seven cognitive domains: Memory, Attention, Flexibility, Problem Solving, Speed of Processing, Math, and Language. The majority of games, however, make demands in multiple cognitive domains (e.g., Processing Speed and Flexibility, or Attention and Memory).

Addition Storm (Math)

Addition Storm is similar to Raindrops (see below) but restricted to addition problems.

Assist Ants (Attention)

Assist Ants exercises divided attention and multiple object monitoring. In this game, users are required to track multiple moving ants and prevent them from colliding with each other or other obstacles by placing raindrop bumpers. Ants continually accrue seeds on their backs while they move around the screen and lose their seeds when they have collisions. There are 10 levels. New obstacles are introduced in the first 4 levels and then found in different combinations after that, giving users some variety in and across levels. The goal of the game is to maximize the number of seeds collected. Reaching a certain threshold number of seeds in a 60 second round will advance the user to the next level.

Birdwatching (Attention)

Birdwatching engages and exercises distributed and divided visual attention and speed of processing. This game challenges the user to identify a centrally presented letter while simultaneously indicating the position of a peripherally presented bird and a varying number of distractor objects. The visual system is challenged further by the introduction of complex backgrounds, picturing natural scenes. The user then engages in a secondary task in which a word must be constructed from the identified letters. The game advances to the next level when the user achieves a criterion level of performance. Each progressive level is marked by shorter presentations of central and peripheral targets and increasing numbers of distractor objects in the periphery.

Brain Shift (Flexibility)

Brain Shift exercises task switching. In this game, two cards are presented on the screen. On each trial, a number and a letter appear on one of the cards. If the stimulus appears on the top card, then the user should indicate “yes” if the number is even and “no” if the number is odd. On the other hand, if the stimulus appears on the bottom card, the user should indicate “yes” if the letter is a vowel and “no” if the letter is a consonant. After a number of correct responses, the challenge is increased by removing the prompt explaining the rules; thus, the user must remember the rules while accurately and rapidly indicating responses. The user is asked to respond quickly and accurately to as many stimuli as they can in a 60 second time period.

Brain Shift Overdrive (Flexibility)

Brain Shift Overdrive is very similar to Brain Shift (see above), but with increased challenge in task switching. In this version, there are four cards, arranged in a grid. If the number and letter appear in the upper left, then the correct response is to click “yes” when the number is even and “no” when the number is odd. If the number and letter appear in the lower left, then the correct response is to click “yes” when the number is odd and “no” when the number is even. The user is looking for a vowel in the upper right and a consonant in the lower right. The user is asked to respond quickly and accurately to as many stimuli as they can in a 60 second time period.

By the Rules (Problem Solving)

By the Rules exercises logical reasoning and working memory. In this game, users must identify the hidden rule in a dynamic card game by indicating whether each revealed card follows the rule. Rules involve features of the pattern depicted on the card, such as color, shape, and number. Exercising mental flexibility and working memory, the user is challenged to formulate hypotheses about what the current rule might be and then dynamically update that hypothesis as new information becomes available. The user is told when they have guessed a particular rule by correctly sorting a certain number of cards consecutively, and when the next rule is being presented. This type of reasoning ability involves the intersection of inductive and deductive reasoning, and in that way mimics the type of decision-making that happens in a wide variety of real-world contexts. Users try to determine as many rules as possible within a limited number of cards.

Chalkboard Challenge (Math)

Chalkboard Challenge exercises quantitative reasoning and calculation. In this game, users are presented with two arithmetic expressions, one on the left and one on the right, and must indicate which value is larger (or whether they are equal). The game rewards speed and accuracy. As users complete trials successfully, the arithmetic expressions become more complex, beginning with single numbers and progressing up to expressions including several integers, operators (addition, subtraction, multiplication, and division), and levels of parentheses. The user is asked to respond quickly and accurately to as many stimuli as they can in 50 seconds plus additional time awarded for correct answers.

Color Match (Flexibility)

Color Match exercises response inhibition and selective attention. This game is based on the Stroop Task, a classic test of semantic interference between color and meaning of words. Each trial shows two cards, each displaying a color word (black, red, blue, or yellow). The text displayed on the right card is presented in one of the four aforementioned colors while the text displayed on the left is presented in black. Users must read the word on the left card and indicate “yes” if the meaning of that word matches the color of the text on the right card and “no” if the color does not match. The user should ignore the meaning of the word displayed on the right card and only pay attention to its color. The goal is to make as many correct responses as possible per 45 second session, and users are additionally rewarded for completing sequential trials correctly.

Contextual (Language)

Contextual exercises reading comprehension and vocabulary knowledge. Each round consists of a short story or article with one passage of text presented at a time. Each passage contains one or more

incorrectly used words. The user must click each incorrect word in the text and then choose a replacement from the sidebar. Game difficulty is adaptively adjusted along two scales, one measuring user success identifying errors (comprehension), and the other their success selecting appropriate word replacements (vocabulary).

Continuum (Language)

Continuum exercises vocabulary knowledge. This game requires users to order 3 or 4 words that vary along a (semantic) continuum according to their order of magnitude (e.g., elder, adult, child). Each game consists of 12 trials. Difficulty (defined by list word frequency, length, and school-grade level) is adjusted trial-by-trial based on accuracy and speed. Game score is based on difficulty level, accuracy, and speed.

Disconnection (Flexibility)

Disconnection is similar to Disillusion (see below) except that the colors and shapes change in each session.

Disillusion (Flexibility)

Disillusion exercises task switching, response inhibition, and selective attention. In this game, users are presented with a grid of connected puzzle pieces, each marked with a shape and color. The user is presented with a single additional piece and must place it on the puzzle. The orientation of that piece indicates whether to match it to contiguous pieces based on the color attribute (if long dimension is along vertical axis) or shape attribute (if long dimension is along horizontal axis). A user is penalized for not matching a piece on the correct attribute. Users must learn the association and then place as many pieces as quickly and as accurately as possible in a 60 second time period.

Division Storm (Math)

Division Storm is similar to Raindrops (see below) but is restricted to division problems.

Eagle Eye (Attention)

Eagle Eye exercises integration of information across the visual field. This game is similar to Birdwatching (see above) but adapts to the user's performance within a session. The user must identify a centrally presented number or symbol while simultaneously indicating the position of a peripherally presented bird in the presence of distractors. The game increases in difficulty by shortening presentation time, increasing visual range, and increasing the number of distractors.

Ebb and Flow (Flexibility)

Ebb and Flow exercises task switching, response inhibition, and selective attention. Each trial consists of a screen full of leaves all pointing in one direction and all traveling in one direction. The leaves are color coded and, depending on the color, the user's task is either to indicate the direction the leaves are pointing or the direction the leaves are moving. The response is a 4-alternative forced choice task (up, down, left, or right). Trials can either be congruous, in which the leaves are pointing in the same direction that they are moving, or incongruous, in which the leaves are pointing in a different direction from the direction of motion. The color of the leaves dictates the rule the user is asked to follow for that trial, with green meaning the user should indicate which direction the leaves are pointing and yellow meaning that the user should indicate which direction the leaves are moving. The leaf color (rule) is

maintained for a random number of trials in order to build up an automatic response. When the leaf color (i.e., rule) is switched, users must then switch their focus to the opposing attribute. Users are asked to respond to as many trials as quickly and accurately as possible in a 60 second time period.

Editor's Choice (Language)

Editor's Choice exercises vocabulary knowledge and fluency. This game involves the recognition of synonyms. On each trial, a list of words is shown with a target word at the top. The rest of the list contains either one or two synonyms of the target word, which the user is supposed to select as quickly as possible. Each game consists of 10 trials. Difficulty level is the school grade level of the words and is adjusted trial-by-trial based on the user's performance. Scoring is based on accuracy and difficulty level.

Face Memory Workout (Memory)

Face Memory Workout is an n-back visual working memory task and is similar to Memory Match (see below). Rather than matching abstract objects, however, users must recognize and match human faces. By responding accurately, users can progress from 1-back to 2-back to 3-back, meaning that users must match the current trial with the trial that appears one trial previously, two trials previously, or three trials previously. Game score is based on speed and accuracy.

Familiar Faces (Memory)

Familiar Faces exercises long-term and short-term memory. This game challenges the user's ability to create associations between visual and verbal information, such as associating a person's name with their face. In this game, the user is asked to remember names and food orders from multiple characters. The user is later asked to recall each character's name and to select their order from a list of options. In order to earn larger tips, the user must recall the names and orders accurately. As the user progresses by answering accurately, the game becomes more complicated, with more characters and more complex orders. Not only does the user need to remember names during a single session, they must also remember names from past sessions, mimicking the real life situation and challenging long term memory. Game score is tied to total tips collected.

Feel the Beat (Attention)

Feel the Beat exercises temporal attention and response inhibition. In this game, the user is presented with a drum pattern and then asked to imitate the pattern 5 times in order to establish a sense of its timing and rhythm. Auditory beats are accompanied by visual cues displayed as pink and blue icons (beats) that pulse sequentially in a clockwise direction around a pair of rings (representing a musical measure). The user must establish and maintain a rhythm that enables them to tap the right keys/buttons at the right times to match the pattern in each trial. The user's timing (how close they are to hitting the right buttons/keys at the right times) are translated into accuracy ratings, and users must achieve at least 80% accuracy in a play session in order to advance to a higher level.

Follow That Frog (Memory)

Follow That Frog exercises spatial working memory. This game challenges users to follow the hops of a frog across a pond filled with lily pads. The game is an adaptive spatial version of the n-back task in which users must indicate where the frog hopped a given number of hops back. This number increases with several successive successful trials and decreases after a mistake. Progress persists across sessions.

This game is a variation on n-back designed to be more interactive than the standard match/no match framework. The basic premise is a follow-the-leader task, where the user must follow the computer-controlled frog and jump on the same lily pads in the same order. In this variation, the n-back value is the number of moves the user is behind the computer-directed frog. Game score is based on accuracy and difficulty level (number of lily pads between the leader and follower).

Fuse Clues (Problem Solving)

Fuse Clues exercises logical reasoning, quantitative reasoning, and calculation. The main goal of this game is to develop the user's ability to recognize quantitative relationships or rules in number series. The game requires users to examine a sequence of numbers and generate a hypothesis about the relationship that exists between them. On each trial, some numbers in the sequence are left blank, and the user is asked to select an appropriate number to go in each blank space. Numbers are represented in the game as fuses that are plugged into an outlet to complete a circuit and charge a battery. A gameplay is composed of 4 trials and users advance adaptively from one trial to the next, moving up or down in level depending on their performance and speed of success. Game score is based on accuracy and level.

Halve Your Cake (Math)

Halve Your Cake exercises quantitative reasoning and calculation. This game challenges users to quickly compare and solve mathematical problems involving fractions that are presented in a variety of visual and symbolic forms. Users have a limited time period to solve as many math problems/comparisons as they can. Each problem represents a step in a recipe, and each completed recipe represents a different level. Problems increase in difficulty through a total of 12 levels, with each level culminating in the unveiling of a new baked creation. Game score is based on speed and accuracy.

Highway Hazards (Speed of Processing)

Highway Hazards exercises processing speed and divided attention. The goal of the game is to react quickly and efficiently to various road obstacles while going around a racetrack. The user's speed increases as they dodge obstacles. Each obstacle is preceded by a warning sign indicating in which of the three lanes of the track it will appear. As the player goes faster, the warning signs appear for shorter amounts of time. Obstacles include rough patches of road and tumbleweeds. Additionally, the user must avoid cars coming in the opposite direction, which can be seen as they approach. Each game lasts approximately a minute and a half to two minutes. At that time, a finish line scene appears, along with a score based on the user's speed and number of obstacles avoided.

Lost in Migration (Attention)

Lost in Migration exercises selective attention and response inhibition. This game utilizes a simple flanker task. Each trial consists of a presentation of five birds pictured in a pattern resembling a flock in flight. The user's task is to indicate the direction of the middle bird. The response is a 4-alternative forced choice task. Trials can be either congruous, where the middle bird is pointed in the same direction as the other birds, or incongruous, where the middle bird is pointed in a different direction. The challenge of response inhibition occurs on the incongruous trials, when the user is tempted to indicate the direction of the majority of birds. The goal is to make as many correct responses as possible per 45 second session. Game score is based on speed and accuracy.

Magic Chance (Math)

Magic Chance exercises quantitative reasoning and calculation. The goal of this game is to analyze and evaluate probability questions presented using virtual cards. A game session includes 5 trials, and in each trial, users are given up to 2 tries to solve the probability question. The user responds to a question by placing cards with pictures of objects onto the table to achieve the desired distribution of object qualities described (e.g., chance of purple or star = 1/3). Object qualities differ in terms of shape and color. Correct answers trigger green check marks, wrong answers trigger red Xs. When two incorrect answers are given within a trial, the trial ends and users are shown an example of a correct answer. The game ends when 5 trials have been completed, either correctly or incorrectly. Difficulty level is adjusted adaptively, based on speed and accuracy. Game score is calculated from accuracy, speed, and difficulty level.

Masterpiece (Problem Solving)

Masterpiece exercises spatial reasoning. In this game, the user rotates and places puzzle pieces to fit together within a puzzle frame. Each game session is composed of 4 puzzles. These puzzles range from two to nine pieces. Score and difficulty level are based on the number of clockwise or counter-clockwise rotation steps used to solve the puzzle in addition to speed. To advance in difficulty and achieve higher scores, the user must respond quickly and make as few rotations as possible to fit all the pieces into the presented puzzle.

Memory Lane (Memory)

Memory Lane is a stylized version of the dual n-back task. The user passes by successive apartment buildings. As they pass by each, a person appears in one of its multiple windows and speaks a letter (presented auditorily). Users must remember where the person appeared (which window) and which letter was spoken a given number of buildings back. Users compare these to those for the current buildings, indicating a location match by pressing the left arrow, a spoken letter match by pressing the right arrow, a match on both by pressing both arrows, and withholding a press to indicate a lack of match. The game challenges memory further by asking users to recall all the letters they heard during the round.

Memory Match (Memory)

Memory Match is a 2-back visual working memory task. Users indicate whether the current symbol matches the one presented two symbols previously. A symbol is presented in a box on the right of the screen. The user indicates whether there is a 2-back match, and then the symbol shifts one position to the left. After the next response, the first symbol slides one more position to the left and is now the to-be-matched target. Initially, the current symbol and the previous two are all visible. After the user starts responding correctly, only the current symbol is visible. Game score is calculated from speed and accuracy.

Memory Match Overdrive (Memory)

Memory Match Overdrive is similar to Memory Match (see above) but provides additional challenge because it is a 3-back task. Users must indicate whether the current symbol matches the one presented 3 symbols previously. Similar to Memory Match, all symbols are initially visible but as the user starts

responding correctly, only the current symbol remains visible. Game score is calculated from speed and accuracy.

Memory Matrix (Memory)

Memory Matrix exercises short-term memory. In this game, users exercise their spatial short-term memory by remembering the location of squares on a grid. The target squares appear briefly and simultaneously at the beginning of a trial. The user must remember the location of all the target squares and click on them after they vanish. Initially, three squares appear and the grid size is 3×3. However, with each correct response, the number of targets is increased by one and the grid size grows. Each time an incorrect response is made, the number of targets is reduced by one and the grid size shrinks. Game score is based on accuracy and level (level increases as the number of target squares increases).

Memory Serves (Memory)

Memory Serves exercises working memory. Playing involves accurately storing incoming information in working memory and continuously updating it as new information comes in. Users assume the role of a bellhop riding up and down in an elevator. On each floor, the bellhop needs to pickup or deliver different types of luggage. The user must keep track of the bellhop's current luggage inventory and only deliver the luggage if it is in the inventory. As the user responds accurately, the bellhop receives more luggage of varying types and elevator stops become briefer, requiring the user to respond more quickly to luggage requests from guests. The game is completed successfully by remembering and dropping off all the correct items to the guests within the time limit. Games last one to three minutes, depending on the difficulty level. Both game score and level are based on the difficulty of the individual deliveries, which depend on the amount and type of luggage to be delivered as well as duration of the elevator stop.

Moneycomb (Memory)

Moneycomb exercises visuospatial working memory. Users must remember the locations of coins hidden within a hexagonal grid (honeycomb) structure. Copper, silver, and gold coins labeled with values 1, 2, and 3, respectively appear in sequence. Users must recall the locations of these coins in the order of their value. Difficulty increases on subsequent trials by increasing the number of coins.

Monster Garden (Memory)

Monster Garden exercises visuospatial working memory. The game challenges users to remember the location of several obstacles that appear only momentarily while navigating the game environment. The game environment is a garden in which obstacles, initially in the form of cartoon monsters, appear briefly one by one. The user must navigate the game environment while avoiding the hidden obstacles to reach a flower. If the user clicks on a space containing an obstacle, a life is lost. The game ends after 3 lives are lost. Levels increase in difficulty by expanding the garden and increasing the number of obstacles that the user must maintain in working memory.

Multiplication Storm (Math)

Multiplication Storm is similar to Raindrops (see below) but restricted to multiplication problems.

Name Tag (Memory)

Name Tag exercises working memory. In this game, users must discover and remember matching pairs of cards within a grid. At the beginning of each session, name tags are displayed that include names

paired with the corresponding faces. During game play, users must match name cards with their corresponding face cards.

Observation Tower (Attention)

Observation Tower exercises short-term memory and integration of information across the visual field. This game challenges users to process briefly flashed information quickly. In the game, a set of numbers is presented for a short time and then disappears, leaving only indicators of the numbers' locations. Users must indicate the order of digits they saw in ascending order. Each digit observed and correctly ordered goes towards constructing a tower. The goal is to recall and correctly order as many digits as possible. Observation Tower adapts to user performance by decreasing presentation time as well as increasing the number of digits to remember as the user demonstrates proficiency. Game score is based on accuracy and difficulty level.

Organic Order (Problem Solving)

Organic Order exercises logical and spatial reasoning. This game requires the user to organize items sequentially based on given rules. On each trial, the user is asked to place a number of different fruits and vegetables into a specific order according to a set of provided rules. On some trials, some items are already placed in specific locations and cannot be moved. Periodically, a set of rules will create a logically impossible ordering for the fruits and vegetables. In these cases, the correct response is to select an 'Impossible' button. Correct answers are met with a check mark and the "correct" sound. Wrong answers receive a red X, the "wrong" sound, and one chance to resubmit. A game consists of 7 trials plus 1 additional challenge trial if all previous trials were answered correctly. As users respond correctly, difficulty increases through more complex rules. A higher score is achieved by reaching more difficult puzzles.

Penguin Pursuit (Speed of Processing)

Penguin Pursuit exercises response inhibition, task switching, and spatial reasoning. This game requires users to guide a penguin through a maze while racing against a computer-controlled competitor penguin. The maze repeatedly rotates its orientation, so that users must mentally recode directions – for example, up becomes left. The user must continue to guide the penguin according to the original directions as quickly as possible. If the competitor penguin reaches the end of the maze first, a life is lost. The game ends after 3 lives are used. The task increases in difficulty by increasing rotation frequency, increasing maze size, and increasing speed of the competitor penguin.

Pet Detective (Problem Solving)

Pet Detective exercises planning and spatial reasoning. In this game, users must pick up pets and return them to their homes within a connected map. Users are limited in the distances they can travel and number of pets they can move simultaneously, and thus must plan a route several steps ahead of time to successfully complete the task. Difficulty increases through the number of pets, distance between homes, and map size. Game score is based on efficiency, speed, and number of pets delivered to their respective homes.

Pinball Recall (Memory)

Pinball Recall exercises spatial working memory. This game requires the user to remember the location of multiple objects and predict a ball's path accounting for those objects. The game presents the user

with a grid and one or more bumpers that have a specific orientation (±45°). The bumpers disappear after a brief presentation time, after which a point on the outside of the grid is highlighted to designate the ball's starting location. The user must then determine the path a ball will travel after emanating from the highlighted point, taking the various bumpers into account (balls ricochet at 90° off bumpers), and indicate which point on the outside of the grid the ball will emerge. Difficulty increases with larger grids and more bumpers. Game score is based on accuracy and difficulty level.

Pirate Passage (Problem Solving)

Pirate Passage exercises planning and spatial reasoning. In this game, the user is asked to draw a path for their ship to a treasure chest marked with an X. The user selects the path one square at a time, while avoiding collisions with other ships moving on their own individual paths, marked by color-coded lines. An attempt fails if the user's ship bumps into another ship and succeeds if they reach the treasure chest. Game score and level increase when fewer moves (squares traversed) are made by the user to reach the treasure chest.

Playing Koi (Attention)

Playing Koi exercises visual divided attention and multiple object monitoring. The goal of the game is to feed all the koi. All the koi fish look exactly the same and swim around the pond. The user must feed each exactly once, which challenges the user to focus on multiple targets simultaneously and follow them throughout their journey across the pond. Lily pads may temporarily obscure koi as they travel. Focus is critical to successfully keep track of all the koi and avoid the distractions of other fish moving around the pond. As performance improves, the task becomes significantly more difficult with more fish. Game score is based on accuracy and difficulty level.

Raindrops (Math)

Raindrops exercises basic arithmetic skills and divided attention in a speeded manner. Arithmetic problems (enclosed in water droplets) fall from the top of the screen, and users must enter the answers before the droplets reach the water level at the bottom of the screen. As the user progresses the difficulty increases, with more complex problems falling more frequently. If a drop hits the water, the water level rises, reducing the amount of time a user has to respond to future drops. The game progresses from simple single-digit addition to multiple digit addition, subtraction, multiplication, and division. Game score is related to total number of problems correctly solved.

Rhyme Workout (Memory)

Rhyme Workout is an n-back task that is similar to Memory Match (see above). Rather than matching exact objects, however, cards with words on them appear, and users must decide whether the word n trials ago rhymes with the current word. Thus, the task engages phonemic processing while simultaneously challenging working memory. Users can progress from 1-back to 2-back to 3-back. Game score is based on speed and accuracy of responses in a limited time period.

Rhythm Revolution (Attention)

Rhythm Revolution exercises temporal attention. Users are presented with a record around which note cues are arranged in a rhythmic fashion. The record rotates, and the user listens to the rhythm for two revolutions. Following this, the user repeats the rhythm with the spacebar for four revolutions. As the user graduates to harder trials, eighth notes, sixteenth notes, triples, and fading notes are incorporated.

River Ranger (Speed of Processing)

River Ranger exercises short-term memory and divided attention. This game requires the user to direct attention to multiple stimuli while remembering which specific stimulus was seen in the previous trial. Users must quickly observe simultaneous stimuli and remember their salient visual properties. On each trial, several animals simultaneously emerge from a river for a brief duration. Users must select which one of the animals had appeared during the previous trial. The goal is to make as many correct responses as possible in a fixed period of time. Each game runs for 1 minute and 30 seconds.

Robot Factory (Flexibility)

Robot Factory exercises response inhibition. In this game, users must respond to the temporary appearance of robot parts on three pedestals and select parts to help complete a robot. Some parts that appear will not fit on the robot, in which case, the pedestal indicates an improper part. Users must then withhold their response and not select that pedestal until the part disappears. The game adapts in real time and, as users progress, parts are introduced more quickly and the frequency of improper parts increases. Game score is tied to speed and accuracy.

Rotation Matrix (Memory)

Rotation Matrix exercises visual short-term memory and mental rotation. This game is similar to Memory Matrix (see above), with the addition of a rotation component that makes the task more challenging. In Rotation Matrix, users are asked to remember the location of squares on a grid. The target squares are presented simultaneously for a brief period of time, and after they disappear, the entire matrix rotates 90 degrees left or right. The user must indicate the location of the target squares in their new locations, which requires them to visualize how the target squares would have moved during the rotation. The task begins with a 3×3 grid and one target square for the user to remember. The grid size and number of target squares increase with correct performance and decrease with incorrect performance. Game score is based on accuracy and difficulty level.

Route to Sprout (Flexibility)

Route to Sprout exercises planning through a series of puzzles where users must move an object (a seed) to the goal. Waypoints are connected by paths, but many waypoints contain obstacles (ladybugs). The user can move objects from waypoint to waypoint, but each waypoint can only hold one object at a time. On each trial, users must move the objects one at a time in order to get their seed to the goal, where it is planted. The goal is to complete the puzzle in the smallest number of moves, as quickly as possible. Route to Sprout increases in difficulty by increasing path complexity.

Skyrise (Attention)

Skyrise is similar to Observation Tower (see above) and exercises short-term memory and integration of information across the visual field. This game challenges users to quickly process briefly flashed information. On each trial, a series of numbers is flashed briefly on a collection of blocks. Users must remember the numbers and click on the blocks on which they appeared in numerical order from lowest to highest. The goal is to correctly identify as many numbers as possible. Blocks with successfully remembered numbers contribute to building a tower. The goal is for users to try to build the tallest tower. Each game consists of 10 trials, and users progress through difficulty levels by correctly identifying all the numbers presented on a given trial. Difficulty is adjusted within each game and is

determined by the number of flashed numbers, their spatial configuration, and visual angle subtended. Game score is based on accuracy and corresponding tower height.

Space Junk (Attention)

Space Junk exercises numerical estimation. It is a subitizing task where users are briefly presented with a number of scattered space-themed silhouettes moving on a black background. The silhouettes disappear and users must indicate how many objects they saw. In this task, the user generally does not have enough time to count the objects on the screen and must instead estimate how many there were. Difficulty is increased by increasing the number of objects and amount of motion.

Space Trace (Flexibility)

Space Trace exercises spatial short-term memory and the generation of new patterns. The goal of this game is to generate as many unique visual patterns as possible. The user is asked to draw 5 lines through a series of presented dots (dots can be used more than once in a pattern). Once 5 lines have been drawn, the design is judged as either unique or repeated, depending on whether the user has drawn this same design this game. Each unique design gains the user points and receives positive feedback. If the design is not unique, the user receives feedback that it is a repeat. The design is then cleared and the user may begin another. The game lasts for 45 seconds, plus bonus time earned for correct designs. Game score is based on the number of unique designs drawn during the time period.

Spatial Speed Match (Speed of Processing)

Spatial Speed Match exercises short-term memory. This game is similar to Speed Match (see below), except that the stimuli on the cards are three circles arranged in a triangle with one circle filled, rather than the shapes used in Speed Match. This challenges spatial discrimination rather than object discrimination.

Speed Match (Speed of Processing)

Speed Match exercises short-term memory. In this game, a stack of cards with shapes on them is flipped one at a time, and the user indicates whether the current card matches the previous card (1-back). Performance is measured by how fast and accurate a user can perform in a fixed timeframe, and the experience is designed such that successful users will increase speed across sessions while maintaining high accuracy.

Speed Match Overdrive (Speed of Processing)

Speed Match Overdrive is an advanced version of Speed Match and exercises working memory. In Speed Match Overdrive, symbols have shape and color attributes, and the user indicates whether the current symbol matches the previous symbol exactly, partially, or does not match. This task challenges the user to respond quickly and accurately. The response is biased toward accuracy by including increasing bonuses for increasingly long chains of correct responses. Game score is based on speed and accuracy.

Speed Pack (Speed of Processing)

Speed Pack exercises spatial reasoning and visualization skills in a physically intuitive task. Users are presented with suitcases filled with objects. One of these objects (a camera) is moveable, and users must position that object such that when the suitcase is folded, none of the objects overlap each other. Trial

complexity is adapted based on performance – more objects and more folds are introduced to users completing trials quickly and accurately. Users are encouraged to pack as many suitcases as possible within a limited amount of time. Game score is based on speed and accuracy.

Splitting Seeds (Speed of Processing)

Splitting Seeds exercises counting and numerical estimation. This game requires using a stick to divide a collection of seeds into two equal parts. On each trial, an even number of seeds and a stick appear on the screen. The seeds cannot be manipulated, so the user must rotate the stick on the board to divide the seeds into two equal parts. Users try to quickly and accurately divide seeds on as many trials as they can within the game duration of 1 minute and 30 seconds. Difficulty is adjusted by increasing the number of seeds presented. Both difficulty level and score are based on speed and accuracy.

Star Search (Attention)

Star Search exercises selective attention. In this game, users must find a single unique object among a field of objects. The objects are shapes with primitive physical characteristics, such as color, orientation, motion, and shading. Sets of other objects share characteristics with the target object to distract users. If users find the unique object quickly, the game increases in difficulty with the introduction of characteristics that are harder to tell apart. For example, the game begins with different shapes with different colors. At higher levels, the shapes and colors are identical, but the target object is rotated in a different direction from that of the distractor objects. The number of objects that share some, but not all, characteristics also increases. Game score is based on speed and accuracy.

Subtraction Storm (Math)

Subtraction Storm is similar to Raindrops (see above) but restricted to subtraction problems.

Taking Root (Language)

Taking Root exercises vocabulary knowledge and verbal fluency. The game features two phases of six trials each, a root definition learning phase and a word composition phase. On each trial of the first phase, a root is given along with three words that include the root. The words can be tapped on to give a definition. On each trial of the second phase, a partial word is given with component roots to fill in. The roots come from a selection of roots learned previously. Level (difficulty) is adjusted based on speed and accuracy. Points are awarded on each trial based on accuracy, speed, and difficulty.

Tidal Treasures (Memory)

Tidal Treasures exercises short-term memory and working memory. In this game, an increasing number of objects wash up on a beach, and in each trial the user must select an object not previously selected. Items include things like seashells, kelp, flotsam, and jetsam that one might expect to find on a beach. More items appear in each trial, taxing the user's memory, until the user selects an object that had been previously selected. As users progress through rounds, difficulty also increases by decreasing the uniqueness and distinctness of objects. The task differs from other working memory exercises by allowing users to choose the objects they wish to remember, which engages other executive functions. Game score is based on the number of unique objects identified.

Top Chimp (Attention)

Top Chimp exercises short-term memory and integration of information across the visual field. In this game, the user briefly sees scattered poker-style chips with numbers on them. The numbers disappear and the user must order the chips in ascending order. Users compete against computer opponents and gauge their own ability by betting different numbers of chips depending on their confidence in beating the opponent. Difficulty is increased by decreasing presentation time and increasing the number of chip numbers to be remembered, as well as increasing the number of chips necessary to bet in order to beat the computer opponent.

Top That (Math)

Top That exercises fast numerical calculation and estimation. In this game, users are asked to quickly calculate and/or accurately estimate prices in order to swap/trade for ever-increasing valued prizes before time runs out. The goal for the user is to end their session with a much higher priced prize than what they started with. As the user trades for higher and higher tiered offers, the game introduces challenges that make the price calculation more difficult to compute in the time given. These challenges include multipliers, addition/subtraction, and percentages. All offers are available only for a limited time, expire, and are replaced quickly. Timing of the offers is staggered. This keeps the pace of the game quick and encourages the player to estimate rather than calculate. The game lasts for 50 seconds. Game score is based on accuracy (selecting a top offer) as well as overall ending offer value and level reached.

Train of Thought (Attention)

Train of Thought exercises divided attention. This game requires users to direct a set of color-coded trains to their destinations. Trains emerge from a tunnel and travel continuously along a branching set of tracks. Users must click switches at the branch points in order to correctly direct the trains to their destinations. Train frequency changes within a session to match a user's performance, increasing with correct deliveries and decreasing with incorrect deliveries. When users perform well, delivering nearly all trains to their correct destination, the user advances in level and the number of stations increases. Score is based on level and number of trains directed to the correct station.

Trouble Brewing (Attention)

Trouble Brewing exercises divided attention. In this game, users manage a coffee shop. Coffee orders are introduced and must be filled by adding the requested ingredients to a cup and then starting the coffee dispenser. Care must be taken to stop the machines at the right time, so that the cups are filled but not overfilled. Users must plan out their strategy, using multiple machines to make use of their time most efficiently, and to serve all customers as quickly as possible. At higher levels, users must manage multiple screens, taking orders on one and executing them on two others. Game score is based on speed and accuracy.

Word Bubbles (Language)

Word Bubbles exercises verbal fluency. A three-letter word stem is presented and users are given 1 minute to enter as many words as possible that begin with that stem. To receive high scores, users must enter multiple words of various lengths ranging from 4 letters to 13+ letters.

Word Bubbles Rising (Language)

Word Bubbles Rising enhances the gameplay of Word Bubbles (see above) by introducing 4 and 5 letter word stems in subsequent rounds, which are shortened to 3 letters after a set time interval. Game score is based on the number of words generated, length of words, and difficulty level.

Word Snatchers (Language)

Word Snatchers exercises vocabulary and verbal fluency. This game involves solving anagrams. On each of 12 trials, the user is presented with the definition of an unknown word and a set of letters. The letters begin to rise, indicating a set time limit to solve the anagram, as the user can no longer guess once the letters reach the top of the screen. The user may request hints but doing so lowers their overall score. The number of letters in the word range from 3 to 10. Difficulty of the word, based on its length and frequency in English, is adjusted trial to trial based on the user's performance. Game score is based on accuracy, speed, and difficulty.

Word Sort (Problem Solving)

Word Sort is similar to By the Rules (see above). However, instead of pictorial items, words must be classified. Word categories can include the number of letters, typeface, starting and ending letter, and semantic relationships.

Supplementary Note 7: FDA Clearance to Market a Digital Therapeutic Using Lumosity Games

On June 13, 2025 the Food and Drug Administration (FDA) granted clearance to market a digital therapeutic based closely on the Lumosity training employed in the present study, aimed at improving attention function in adults with ADHD (K243729 on FDA 510(k) Clearances page). The name of the digital therapeutic is Prismira and its indications for use are as follows:

" Prismira is a digital therapeutic indicated to improve attention function in adults ages 22-55 years old with primarily inattentive or combined-type Attention Deficit and Hyperactivity Disorder (ADHD). Patients who engage with Prismira demonstrate improvements in a digitally assessed measure of sustained and selective attention, Test of Variables of Attention (TOVA), and may not display benefits in typical behavioral symptoms, such as hyperactivity. Prismira should be considered for use as part of a therapeutic program that may include clinician directed therapy, medication, and/or educational programs, which further address symptoms of the disorder." (Decision Summary)

This clearance was supported by a large randomized, double-blind, sham-controlled clinical trial (NCT05296473, pre-registered on ClinicalTrials.gov), which demonstrated that Lumosity games can elicit meaningful attentional improvements in this population. The present study extends these findings by providing real-world evidence that casual training under naturalistic conditions—using a digital health technology designed for the general public—may similarly improve attention in adults with ADHD.
